# Caregivers’ treatment-seeking behaviors and predictors of whether a child received an appropriate antimalarial treatment: a household survey in rural Uganda

**DOI:** 10.1186/s12879-016-1815-5

**Published:** 2016-09-06

**Authors:** Rosemin Kassam, Richard Sekiwunga, John B. Collins, Juliet Tembe, Eric Liow

**Affiliations:** 1 School of Population and Public Health, Faculty of Medicine, University of British Columbia, 2206 East Mall, Vancouver, V6T 1Z3 BC Canada; 2 Child Health and Development Centre, School of Medicine, Makerere University, P.O. Box 7062, Kampala, Uganda; 3 Department of Educational Studies; Faculty of Education, University of British Columbia, Vancouver, V6T 1Z4 BC Canada; 4 The Islamic University, Mbale, Uganda

**Keywords:** Malaria, Treatment-seeking, Behavior, Children, Knowledge, Management, Access, Predictors, ACT, Uganda

## Abstract

**Background:**

This study responds to a rural community’s concern that, despite national initiatives, malaria management in young children falls short of national guidelines in their district. This study aimed to: (1) describe caregivers’ treatment-seeking behaviors in the rural district of Butaleja, (2) estimate the percentage of children who received an *appropriate* antimalarial, and (3) determine factors that maximized the likelihood of receiving an *appropriate* antimalarial. *Appropriate* antimalarial in this study is defined as having received only the Uganda’s age-specific first-line malaria treatment for uncomplicated and severe malaria during the course of the febrile illness.

**Methods:**

A household survey design was used in 2011 to interview 424 caregivers with a child aged five and under who had fever within the two weeks preceding the survey. The survey evaluated factors that included: knowledge about malaria and its treatment, management practices, decision-making, and access to artemisinin combination therapy (ACT) and information sources. Bivariate analysis, followed by logistic regression, was used to determine predictors of the likelihood of receiving an *appropriate* antimalarial.

**Results:**

Home management was the most common first action, with most children requiring a subsequent action to manage their fever. Overall, 20.9 % of children received a blood test, 68.4 % received an antimalarial, and 41.0 % received an ACT. But closer inspection showed that only 31.6 % received an *appropriate* antimalarial. These results confirm that ACT usage and receipt of an *appropriate* antimalarial in Butaleja remain well below the 2010/2015 target of 85 %. While nine survey items differentiated significantly whether a child had or had not received an *appropriate* antimalarial, our logistic regression model identified four items as independent predictors of likelihood that a child would receive an *appropriate* antimalarial: obtaining antimalarials from regulated outlets (OR = 14.99); keeping ACT in the home for future use (OR = 6.36); reporting they would select ACT given the choice (OR = 2.31); and child’s age older than four months (OR = 5.67).

**Conclusions:**

Few children in Butaleja received malaria treatment in accordance with national guidelines. This study highlighted the importance of engaging the full spectrum of stakeholders in the management of malaria in young children - including licensed and unlicensed providers, caregivers, and family members.

**Electronic supplementary material:**

The online version of this article (doi:10.1186/s12879-016-1815-5) contains supplementary material, which is available to authorized users.

## Background

Malaria remains an important health challenge worldwide, with an estimated incidence of 219 million infections and approximately 660,000 deaths reported in 2010 [1]. Nearly 90 % of these deaths occurred in sub-Saharan Africa (SSA), of which 91 % were in children under the age of five [1]. Children between three months and five years of age are most susceptible to adverse events from malaria due to waning of their natural immunity from maternal antibodies and an acquired immunity that has not fully developed [2]. Among those who survive, many are left with persistent anemia, impaired brain function, and/or paralysis, all of which hamper physiological and cognitive development.

Country-level data for 2010 indicate that 80 % of malaria cases in the World Health Organization (WHO) African region occur in just 17 countries, with Uganda being among the six most burdened countries [3]. Though a downward trend in the burden of malaria for children under five has been observed in select regions of SSA, limited regional data do not yet support this trend for Uganda [3–5]. In Uganda, where a large majority of the population lives in high transmission areas, the social and economic burden of acute malaria and asymptomatic parasitemia on families and governments is equally staggering [6]. Families commonly incur out-of-pocket expenses when seeking antimalarial treatment for their children, as well as loss of income from missed work days [7].

In 2004, artemisinin combination therapy (ACT) became the first-line malaria treatment for children older than four months, with artemether-lumafantrine (Coartem®) as the first-line option and artesunate plus amodiaquine as an alternative, and quinine as the second-line treatment [8, 9]. The Uganda national case management guidelines were revised in 2005 and 2006 to include the new treatment policy, and provisions were made to distribute Coartem® to government and private-not-for-profit health facilities for dispensing to communities free of charge [8]. Additionally, Uganda introduced a number of interventions in an effort to bring diagnostics and treatment with ACTs closer to the community [10]. At the time of this study, the Uganda Ministry of Health approved two major national programs: the Integrated Community Case Management Program (iCCM) in mid-2010, and the Affordable Medicines Facility – Malaria (AMFm) in Spring of 2011 [10].

Despite such initiatives, national and regional data in Uganda suggest that the use of first-line antimalarials in children under five years of age remains well below the national 2010 and 2015 targets of 85 % [8, 11]. There is, however, limited district-level data, particularly in non-surveillance areas, to inform how closely practices within different rural districts align with regional and national data. Additionally, across Uganda, there is a paucity of research investigating the extent to which childhood malaria is managed using only first-line treatments.

This current study responds to a rural community’s concern with the under-use of effective antimalarials in children five and under during presumed malaria episodes in the district of Butaleja. As part of a large initiative to define sustainable public health programs to improve malaria case management in Butaleja, this study aimed to: (1) describe caregivers’ overall treatment-seeking behaviors, (2) estimate the percentage of children who received an *appropriate* antimalarial, and (3) identify factors that maximized the likelihood of receiving an *appropriate* antimalarial. *Appropriate* antimalarial in this study is defined as having received only the Uganda’s age-specific first-line malaria treatment for uncomplicated and severe malaria during the course of the febrile illness. These objectives were assessed across the district and within different regions of the district.

## Methods

### Study design

A cross-sectional household survey was interviewer-administered with caregivers of children five and under in Butaleja District, Uganda, following a recent fever episode that was presumed to be malaria. The study’s fieldwork was conducted over the two-month period of June and July 2011. Ethics approval for the project had been previously.

### Setting

Formerly part of Tororo District, Butaleja was established as an independent district in 2005 and named after its main town of Butaleja where district headquarters are located [12]. Butaleja District is situated in eastern Uganda, bordered by Budaka District to the north, Mbale to the east, Tororo to the south east, Bugiri to the south, and Namutumba to the west (Additional file 1: Figure S1) [12, 13]. Butaleja’s administrative structure consists of 10 sub-counties (mostly rural) and two town-councils (designated urban centers) [12]. At the time of this study, Butaleja had 66 parishes and 397 villages (K. Mweru, MD, written communication, April 2011). The major ethnic group is the Banyole tribe, representing approximately 66 % − 85 % of the population, followed by the Bagwere tribe at about 5 %, the Jopadhola tribe at 3 %, and other ethnicities (Basoga, Bagisu, Iteso, Baganda, Karimojong, Banyankole, Acholi, etc) making up less than 2 % [12, 13]. The predominant language spoken across the district and by all tribes is Lunyole. The three largest religious groups include Protestants (53 %), Muslims (30 %), and Catholics (17 %) (K. Mweru, MD, written communication, April 2011). A large majority of the people are subsistence farmers, with poverty being a district-wide phenomenon [14]. Life expectancy in 2008 was estimated at around 47 years, and mortality rate for children under five in 2006 was 152 per 1000 live births. Malaria was the single highest ranked cause of morbidity in the period 2007–2009, with about eight in every 10 persons experiencing malaria/fever [12].

The public health infrastructure in Uganda is stratified into four levels: by district, sub-county, parish, and village [15]. The following description is informed, in part, by documents from the Regional District Office of Butaleja (K. Mweru, MD, written communication, April 2011). At the highest level, there is one public hospital located in Busolwe Town-Council, providing outpatient, inpatient, surgical, and community health services. At the next lower level are 11 Health Centre IIIs located in eight sub-counties that are staffed by medical clinical officers, laboratory assistants, nurses, midwifes, and nursing assistants. These centres are regulated to administer and dispense oral and injectable quinine, ACT, and sulfadoxine/pyrimethamine. At a still lower level are the Health Centre IIs, which operate at the parish level. At the time of this study, there were 11 such centres located in select parishes across seven sub-counties. Health Centers II are staffed by nurses and nursing assistants, and regulated to provide a more limited range of care, including administration and dispensing of oral and injectable quinine, artemisinin injection, ACT, and sulphadoxine-pyrimethamine. At the lowest level, Health Centres I exist as informal structures consisting of community volunteers (also known as village health teams or community health workers - CHWs). Their role is to provide basic health services at the community-level, such as the distribution of ACT, the provision of health education advice, and referral to higher level health centers. While it is advocated by the ministry of health that every sub-county have at least one Health Centre III and every parish have one Health Centre II, at the time of this study the district fell short of attaining this goal.

In 2006, the Uganda National Malaria Control Program introduced a policy to provide the first-line antimalarial ACT cost free from all levels of the health system (levels I to hospitals), supplied as the Coartem® brand [16]. This formulation is composed of a fixed combination of 20 mg of artemether and 120 mg of lumefantrine, supplied in pre-packed weight- and age-specific forms. However, at the time of this study, Coartem® had yet to be distributed to CHWs.

Butaleja is also serviced by private outlets where other ACT brands can be purchased for a fee. The district, however, has no pharmacies, and the few licensed drug shops are mainly located in town centers and market areas. Accordingly, unlicensed private vendors who do not have formal training in the management of malaria constitute the large majority of private outlets in villages across Butaleja (R Kassam, E Liow, R Sekiwunga, Unpublished work, August 2011).

### Participants

Household surveys were carried out with caregivers who met the following inclusion criteria: (1) they had at least one child five years or younger who had been febrile within the last two weeks, (2) they were the primary care provider for the child (which included supervision, bathing, and feeding), (3) they resided within Butaleja District at the time of the study, (4) they spoke the common district dialect – Lunyole, (5) they agreed to participate, and (6) they willingly signed the consent form using thumbprint or written signature. Survey questions about current practice were asked in reference to the youngest child with fever (referred to as the “index child”). A household was excluded if its children had no fever within the two weeks prior to the survey, or if the child’s fever was confirmed by a qualified health professional to be unrelated to malaria. The presence of fever was determined by the caregiver, and for a large majority this was detected by touching the skin of the affected child.

### Sampling

The 2002 national census recorded the population of Butaleja District to be approximately 157,475, with an annual growth rate of 3.3 % [12]. Based on this census, it was estimated that by 2010 the population would stand at approximately 206,300, and be comprised of about 41,240 households and 44,300 children under age five [12]. Assuming that every second household had a child of five years and under, the number of households with the target population was estimated at 20,620. The Raosoft sample size calculator showed that a representative sample of that number of households at a 5 % margin of error with a 95 % confidence level required 380 households to be interviewed [17].

The sampling frame for the study covered all 10 sub-counties and two town-councils. Since the town-councils were geographically situated centrally within the district and shared similar urban characteristics, such as greater access to local amenities including licensed drug shops and transportation, they were considered as a unit for sampling purposes. A purposive multi-step sampling process was used to ensure representation of all 10 sub-counties, varying household counts in different villages, different religious denominations, dominant tribes, and proximity to a government health center. In total, 35 different villages were sampled from 27 of Butaleja’s 66 parishes. Sampling at the household level used a simple random process to avoid self-selection. With the help of the local leaders, a central point at each village level was determined and each village was divided into four quadrants using natural boundaries. Since no listing of households with children five years and under was available, the first household in each of the quadrants was selected by a dice throw, screened for eligibility, and recruited if the inclusion and exclusion criteria were met. Subsequently, every second household was visited and assessed for eligibility. If no caregiver was available or if the household did not meet the inclusion and exclusion criteria, then the immediate next household was visited and assessed for eligibility. The process was repeated quadrant-by-quadrant until 12–13 households within each village were recruited. In total 424 eligible households were recruited using this method.

### Research assistant training and deployment

The survey was individually administered in the local language of Lunyole by one of seven research assistants recruited from Butaleja District. The research assistants were recent graduates from a Ugandan university with some field experience and fluency in verbal and written English and in the spoken local dialect. Prior to conducting the survey, all seven research assistants participated in a structured two-week training program consisting of face-to-face large and small group work and field-based exercises. During the training, participants were briefed on the study’s goal and objectives, the household recruitment process, and the survey protocol. The purpose of each survey question along with its response format was discussed in detail. The survey delivery process (first in English, then in Lunyole) was practiced in small groups and rehearsed in the field under the supervision of the study team, and each participant’s ability to elicit the necessary information in an unbiased fashion was assessed. Because Lunyole is not a written language, questions from the English version of the survey were translated verbally during the training using group discussions and consensus supported by local members of the research team who were fluent in both English and Lunyole.

### Instrument development

A survey instrument was developed to assess caregivers’ treatment-seeking behaviors for the management of malaria in young children [see Additional file 2]. A literature review, existing survey instruments, measurement experts, and malaria content experts informed the structure and content of the survey. The final survey included 160 questions consisting of: (1) interviewer instructions, (2) research assistants’ post-interview remarks, (3) household identification, (4) index child’s disease presentation, (5) caregiver and household characteristics, and (6) interview questions. These questions addressed: (1) knowledge about malaria and its treatment, (2) access to ACTs, (3) management of the most recent fever episode in reference to the index child, (4) malaria-related practices, (5) decision-making processes, and (6) information sources accessed. Each question was carefully worded, its order of presentation within the survey was examined, and its response format was keyed carefully to the intent of each question. In order to engage participants on the issue of malaria from the outset, personal and demographic questions were deferred to the end of the survey. Responses were recorded free-form with answer checklists keyed to the questions and a range of word scales. For example, these included dichotomous scales such as: yes/no; true/false, and male/female; present/absent scales such as: no problem/yes problem; and easy/difficult scales for questions about various problems, ease of accomplishing various tasks, or ease of access to a public health facility. Additionally, caregivers identified various medications they obtained for the index child by pointing to photographs printed on laminated posters of all anti-malaria medicines, antibiotics, antihelmetics, analgesics, and antipyretics available within the district.

Prior to administration, the survey was pre-tested with the district key informants to confirm appropriateness of the questions and to ensure the use of culturally suitable terminology. For example, “malaria” and “omusuja” are terms frequently used interchangeably in the district to refer either to fever as a stand-alone symptom (referring to a hot body), or to a variety of illnesses with fever as a common symptom. Similarly, “doctor” and “health professional” are expressions commonly used to refer to anyone who provides treatment. Thus, at the recommendation of key informants, the combined term of “omusuja gwesena” was used to ensure information collected would be about fever brought by mosquitos. For questions relating to health professionals, descriptions of premises where they worked as well as their dress codes were also collected to distinguish between the roles of providers.

### Data collection

Following informed consent from primary caregivers, data were collected through household interviews. Quality control measures were introduced throughout the collection phase to ensure integrity of the data. These measures included a review of the final survey documents for completeness, spot checks by randomly visiting households and asking caregivers about their experiences with the survey, and cross-checks of the completed surveys with the audio recorded interviews for accuracy and completeness. On a day-to-day basis, this process was supported by the local research team consisting of the District Medical Health Officer - who served as the study’s site manager, a retired District Health Educator - who served as the field coordinator, and a behavioral scientist from Makerere University - who was involved with the study design and oversight. Research Assistants were provided regular feedback and, when necessary, caregivers were revisited to clarify information. Survey question data were transcribed from completed survey documents onto Excel spreadsheets for cleaning and verification, then subsequently transported into SPSS® 21.0 for Windows for analysis.

### Measures and analysis

The study’s first objective was to provide an overview of caregivers’ treatment-seeking behavior when managing a child’s malaria episode. As such, frequencies of survey question responses provided a first overview of results.

The second objective of the study was to quantify the percentage of children who received an *appropriate* antimalarial vs. those who did not. A*ppropriate* antimalarial in this study is defined as having received only the Uganda’s age-specific first-line malaria treatment for uncomplicated and severe malaria during the course of the febrile illness (K. Mweru, MD, written communication, April 2011) [9]. For children younger than four months, this included receiving quinine (oral or injectable) or artesunate (injectable or rectal) therapy. For children four months and older, an *appropriate* antimalarial was defined as receiving any ACT and/or artesunate (injectable or rectal) or quinine (injectable). For this study, all ACTs were considered as legitimate first-line treatment. Simply asking a caregiver whether the child had “received an antimalarial” allowed too much latitude for incorrect information, inappropriate combinations, misidentified drugs, or simply lack of knowledge. Therefore, an audit of all medications reported to have been given to manage the current fever episode informed whether the *appropriate* antimalarial criteria had been met for each individual child. In contrast, *ACT usage* in this study followed the definition of Fink et al., with *usage* acknowledged if any of the medicines taken during the febrile illness was an ACT [18]. Frequency of an *appropriate* antimalarial received was calculated for the whole of Butaleja District and for each of its sub-counties and town-councils.

The last objective of the study was to identify whether the likelihood of a child receiving an *appropriate* antimalarial could be determined from caregivers’ answers to select survey questions, including questions about caregivers, index child, and household demographic characteristics. Initially the focus was more on the content of the survey questions than their answers, with approximately 160 different questions inspected for suitability for inclusion to assess whether the likelihood of a child receiving an *appropriate* antimalarial could be determined from caregivers’ answers to these questions. Bivariate analyses, using chi-square test (*χ*^2^) followed by eta-squared (η^2^), then examined approximately 125 questions to determine if they could distinguish whether or not a child received an *appropriate* antimalarial. During data cleaning and verification, certain free-form or complex responses were recoded into simpler forms (Y/N; present/absent; mentioned/not mentioned; etc.) to allow more direct analysis of correlates of receiving an *appropriate* antimalarial or testing for differences among various sub-counties. Questions were retained if: *χ*^2^ test demonstrated associations at or better than *p* < .05 statistical significance, η^2^ of 2.0 % or more (hence accounting for unexpectedly large fractions of variance), or there was a strong conceptual reason for further examining specific questions. Subsequent binary logistic regression tested whether surviving questions-in-combination increased predictability. Examination of the data prior to analysis ensured all predictor variables met the dichotomous level of measurement and that multicollinearity would not present a significant problem.

## Results

### Respondents

A total of 424 interviews were completed with primary caregivers from households across Butaleja District, representing all 10 sub-counties and two town-councils. All caregivers who met the inclusion criteria agreed to participate. Complete information was provided for 399 children, four surveys were partially completed because caregivers had to attend to other responsibilities, and one had missing demographic information. However, all surveys were retained and analyzed for all possible information that was present. Tables 1, 2 and 3 summarize caregivers’, index children, and household characteristics. As shown in Table 1, the study sample adequately reflected the educational, occupational, tribal, and religious diversities within the district.

**Table 1:** Demographic characteristics of caregivers

Caregiver characteristics	Mean	SD	Caregiver characteristics	Number	Percent
Age (*n* = 406)	31 years	10	Currently Working for a Wage (*n* = 420)		
			No	393	(93.6)
	n	(%)	Yes	27	(6.4)
Gender (*n* = 420)			Work Done in Last 12 Months (*n* = 420)		
Female	361	(86.0)	Yes	347	(82.6)
Male	59	(14.0)	No	73	(17.4)
Relationship to Head of Household (*n* = 418)			Current Occupation (*N* = 419)		
Wife	310	(74.2)	Peasant farmer	351	(83.8)
Household head	72	(17.2)	Housewife	35	(8.4)
Daughter/son	15	(3.6)	Petty trade/unskilled laborer	16	(3.8)
Daughter-in-law	10	(2.4)	Professional/shop keeper	7	(1.7)
Parent	5	(1.2)	Other	10	(2.4)
Husband	3	(0.7)	Tribe (*n* = 419)		
Sibling	3	(0.7)	Banyole	312	(74.5)
Relationship to Index Child (*n* = 424)			Bagwere	55	(13.1)
Mother	335	(79.0)	Bagisu	18	(4.3)
Father	46	(10.8)	Basoga	16	(3.8)
Grandparent	31	(7.3)	Jopadhola	9	(2.1)
Household head	9	(2.1)	Itseso	4	(1.0)
Aunt	3	(0.7)	Other	5	(1.2)
Part of the Household (*n* = 423)			Religion (*n* = 424)		
Yes	422	(99.8)	Protestant	211	(49.8)
No	1	(0.2)	Muslim	139	(32.8)
Highest Level of Education (*n* = 419)			Catholic	57	(13.4)
None	73	(17.4)	Born Again Christian	10	(2.4)
Primary incomplete	228	(54.4)	7th Day Adventist	3	(0.7)
Primary complete	48	(11.5)	Other	4	(0.9)
Secondary incomplete	54	(12.9)			
Secondary complete	7	(1.7)			
Post-secondary (technical/ University)	9	(2.1)

**Table 2:** Demographic characteristics of index children

Index child characteristics	Mean	SD
Age (*n* = 408)	22 months	16
	n	(%)
Gender (*n* = 424)		
Female	214	(50.5)
Male	210	(49.5)
Birth order (*n* = 423)		
Youngest	410	(96.9)
2nd Youngest	12	(2.8)
3rd Youngest	1	(0.2)

**Table 3:** Demographic characteristics of households

Household characteristics	Number	Percent
Caregiver’s Perspective of Distance to Nearest Public Health Facility (*n* = 420)		
< ½ Mile	14	(3.3)
½ to < 1 Mile	22	(5.2)
1-5 Miles	308	(73.3)
> 5 Miles	68	(16.2)
Don’t know	8	(1.9)
No. Rooms in this Household (*n* = 419)		
0	6	(1.4)
1	225	(53.7)
2	107	(25.5)
3+	81	(19.3)
No. People Usually Sleep in this Household (*n* = 419)		
2-4	146	(34.8)
5-7	202	(48.2)
8+	71	(16.9)
House Structure (*n* = 421)		
Semi-permanent (Mud)	227	(53.9)
Permanent (Brick)	193	(45.8)
Uniport (Tin House)	1	(0.2)
Type of Dwelling (*n* = 415)		
Independent	316	(76.1)
Shared	85	(20.5)
Muzigo	11	(2.7)
Other	3	(0.7)
Type of Toilet Facilities (*n* = 419)		
Traditional Pit/ Latrine	389	(92.8)
Other	23	(5.5)
Bush/filed/forest	7	(1.7)
Type of Fuel Used (*n* = 420)		
Firewood	408	(97.1)
Charcoal	11	(2.6)
Electricity	1	(0.2)
Transportation Owned (*n* = 420, Multiple Responses)		
Bicycle	258	(61.4)
None	155	(36.9)
Motorcycle	21	(5.0)
Motor vehicle/Canoe/Boat	8	(1.0)
Communication Owned (*n* = 419, Multiple Responses)		
Radio	265	(63.2)
Mobile phone	200	(47.7)
None	104	(24.8)
Television (TV)	11	(2.6)
Main Source of Information (*n* = 424, Multiple Responses)		
Word of mouth	312	(73.6)
Radio	312	(73.6)
Mobile phone	53	(12.5)
None	13	(3.1)
Other (TV, print media/mail/posters)	12	(2.8)

### Caregivers’ practices and management strategies

Four themes relating to caregivers’ practice and malaria management strategies were evaluated, with three of the four themes relating to the index child’s current episode of fever: (1) first action taken by caregiver, (2) subsequent action taken by caregiver, (3) care given to the index child over the course of the illness in the last two weeks, and (4) households’ general practices. Additional file 3: Figure S2 and Additional file 4: Figure S3 illustrate select first actions taken by caregivers, and Additional file 5: Figure S4 and Additional file 6: Figure S5 illustrate those taken during the course of the child’s illness.

#### First action care for the index child

Doing nothing (or only praying) was reported by very few caregivers (4.5 %). Almost three-quarters of index children (73.6 %) received some form of action within 6 h of their caregivers noticing the first symptoms and 14.2 % between 6 and 24 h. Only 2.8 % waited more than 24-h to receive any action. No information was provided for the remaining 5.0 % of the children. In total, 556 actions were provided by 424 caregivers, for an average 1.3 first actions per child. Home management was the most common first action reported by at least 72.6 % of caregivers. Home management was defined as any care initiated from the home setting with resources located within and around the household, such as home remedy (home-stock medicines and physical supportive care) and traditional herbs. Physical supportive care included non-pharmaceuticals, such as bathing, sponging, and giving tea or water for hydration. About three-fifths (63.9 %) of caregivers reported using only one type of option: medicines, supportive care, or traditional herbs; about one-third (29.5 %) used two of the three options; and a small proportion (3.1 %) used all three types of options.

Overall, for the first action, 26.4 % of caregivers reported using traditional herbs, 38.2 % used supportive care, and just under two-thirds (62.3 %) gave some form of medicine. Almost half (48.8 %) of caregivers obtained medicines from an external source and one-third (29.9 %) used medicines from their home-stock. Approximately one-fifth (18.4 %) reported giving medicines from two sources (home-stock and an external source). The three most common classes of medicines given in various combinations included antipyretics (54.7 %), antimalarials (24.3 % gave one antimalarial and 2.6 % gave two antimalarials), and antibiotics (12.7 %). Of the 114 children who received any antimalarial, about one-quarter (26.3 %) received ACT, one-sixth (16.7 %) received quinine, and almost three-fifths (58.8 %) received other antimalarials. For the first action, usage of a first-line antimalarial among the 424 children was 15.1 %, and 7.1 % for ACT.

#### Care over the course of the index child’s illness (within last two weeks)

Just over five-sixths (86.3 %) of index children required a subsequent action to manage their fever. About two-thirds (69.3 %) of caregivers reported seeking care from a trained health professional (a doctor, nurse or medical attendant), with 72.1 % of these visits occurring at public health facilities. Caregivers were 2.3 times more likely to seek care from an external source if they perceived their child’s symptoms to be associated with severe malaria; although, no statistically significant relationship was found between caregivers’ distinction of mild versus severe malaria and whether a child received the first-line antimalarial.

Fewer than one-quarter (20.8 %) of caregivers reported the child to have received a blood test when they presented with the child at an external outlet, although this study was unable to confirm the purpose of these tests. Of those children who were tested, about three-quarters (78.4 %) of them were carried out at public health facilities (representing 32.6 % of children seen at public health facilities for assessment of their fever), 14.8 % at a private clinic, 9.1 % at a drug shop, and one was administered by a CHW. Children who were reported to have received a blood test were no more likely to receive an *appropriate* antimalarial than those who had not received a test.

Of the 424 children, nearly a third (31.6 %) received no antimalarial whatsoever, 31.6 % received an *appropriate* antimalarial, and 36.8 % received subordinate antimalarials. Whether children received an *appropriate* antimalarial or not, across all households surveyed, usage of ACT (41.0 %) or of an age-specific first-line antimalarial (42.0 %) during the course of the illness occurred for fewer than half the children. Two categories of subordinate antimalarial treatments were evident: (1) antimalarial treatments that did not include ACT (26.4 %), and (2) misuse of ACT itself (10.4 %). Examples of the non-ACT antimalarial treatments (used alone or in combination) included quinine (21.7 %), chloroquine (17.9 %), and other antimalarial such as mefloquine and sulphadoxine-pyrimethamine (1.9 %). Misuse of ACTs included administration of ACTs in combination with inferior antimalarials for children over four months (7.3 %) or mis-prescription for infants under four months (2.1 %).

Three-quarters (75.1 %) of the 290 children who received an antimalarial did so within 24-h of presenting with their first symptoms. While most caregivers (94.1 %) who were surveyed expressed that it was their preference to obtain an antimalarial from a public health facility, in fact antimalarials were obtained from both public and private outlets: 61.3 % from public health facilities, 55.8 % from private sector outlets (18.5 % from private hospitals/clinics and 37.3 % from drug shops that were predominantly unlicensed), and 1.4 % from CHWs. Collectively, a large majority (89.7 %) of caregivers who obtained an ACT sourced it from a licensed outlet (public health facility, CHW, or private hospital/clinic), with most (77.0 %) obtaining it from public health facilities. However, of all those who visited a public health facility, only three-fifths (63.0 %) of children received an ACT and two-fifths (43.2 %) received an *appropriate* antimalarial. Nearly four-fifths (81.5 %) of caregivers used only one source for obtaining their antimalarial and fewer than one-fifth (18.5 %) reported using two sources.

#### General household practice

Only one-third (32.8 %) of caregivers believed ACT was easy to find within their community and even fewer (16.5 %) believed ACT was affordable. It was common practice for caregivers to keep an antimalarial medicine in the home for future use in case their child got sick, with a little over half (55.5 %) of caregivers claiming to have kept an antimalarial in the home over the past six months. All ACTs given as part of the first action were given from households’ home-stock medicines. Whether caregivers believed ACT was easy to find or was affordable made no difference to whether the child actually received an *appropriate* antimalarial. However, children of caregivers who knew that ACT was the government’s recommended antimalarial were more likely to receive an *appropriate* antimalarial than those who knew nothing about government recommendations (41.6 % vs 26.6 %, *p* < .002).

### Caregivers’ knowledge

Four themes related to knowledge were evaluated: (1) knowledge about malaria and its cause, (2) knowledge about the management of uncomplicated malaria, (3) knowledge about national policies regarding management of malaria, and (4) preparedness to respond adequately to an acute episode of malaria. Expanding on a classification system by Bartolini and Zammarchi, all symptoms mentioned by caregivers were individually analyzed and clustered into the following five symptom-based categories: (1) subtle warnings signs of a child not being well, (2) early phase malaria symptoms, (3) classical symptoms, (4) severe symptoms, and (5) non-malaria symptoms [19].

#### Knowledge about malaria – its symptoms and cause

Just over half (55.7 %) of the caregivers expressed certainty in their ability to recognize malaria. When asked what symptoms were associated with malaria, caregivers on average listed four different symptoms, and most were indicators of the classical and early phase symptoms associated with malaria (94.8 % and 94.6 %, respectively). A majority (80.2 %) of caregivers reported fever as a sign of malaria, with three-quarters (74.3 %) stating they were very certain they would be able to diagnose fever. However, only 14.6 % of caregivers recognized chills to be associated with malaria. While early phase symptoms collectively were mentioned most frequently, caregivers’ knowledge about the individual symptoms was low, with only 31.1 % mentioning diarrhea, 28.1 % malaise/weakness, 26.2 % vomiting, 5.0 % headache, and 3.6 % pain. Similarly, a small portion (20.3 %) mentioned symptoms associated with severe malaria, with the three most common being: inability to eat/drink/breastfeed (8.7 %), convulsions (5.4 %), and rapid breathing (2.8 %). There were common misconceptions, even among those who had reported correct symptoms, with 60.8 % of caregivers linking runny nose and sneezing with malaria, and 36.1 % associating all types of cough with malaria. When asked to state one main symptom of malaria, caregivers were generally accurate in reporting an actual symptom of malaria, although these were not always aligned with what the medical community considers main (i.e., fever). Two-thirds (62.0 %) of caregivers reported legitimate indicators of malaria: 43.4 % listed one of the two classical symptoms (fever 40.3 %, chills 3.1 %), 13.0 % listed one of the early phase symptoms (diarrhea 4.2 %, malaise 4.2 % and vomiting 2.6 %), and 9.2 % listed subtle symptoms of malaria. However, almost one-third (38.0 %) of caregivers reported symptoms not clinically associated with malaria. Regarding the primary cause of malaria, only three-quarters (78.1 %) of the caregivers mentioned mosquito bites, 2.8 % mentioned a surrogate for mosquito bites such as environmental factors associated with proliferation of mosquitoes, and 19.1 % were misinformed, mentioning causes such as change in season (6.5 %) and ingestion of certain foods and fluids like drinking dirty water, or eating dirty, cold, or certain types of foods (4.8 %). A small percentage (5.4 %) reported not knowing the cause.

#### Knowledge about antimalarial medicines

Most caregivers (95.3 %) believed an antimalarial should be started within 24-h of noticing fever. In terms of most effective medicines (which cured the best), about four-sixth (68.2 %) of caregivers cited an actual antimalarial medicine as being the most effective, and one-quarter (23.1 %) acknowledged not knowing or citing a range of other class of medications (8.7 %) including antibiotics and antipyretics/analgesics. Examples of antimalarials reported included: ACT (35.1 %), quinine (17.7 %), and chloroquine (13.9 %). A similar fraction of caregivers said they would select for their children the very antimalarials they listed for the best cure if given the choice (ACT 32.1 %, quinine 16.6 %, and chloroquine 15.2 %), but 16.1 % mentioned they would select a non-antimalarial such as antipyretic/analgesic (10.4 %) and antibiotic (2.6 %), and 18.6 % stated they did not know what to select.

In terms of least effective antimalarials, 42.7 % reported not knowing, 24.5 % stated antipyretic/analgesic, 16.3 % mentioned antimalarials other than ACT (chloroquine 9.7 % and quinine 5.0 %), 5.2 % stated ACT, and the rest stated a range of other drug classes including antibiotics. As a last-choice antimalarial, only 8.1 % mentioned an ACT, one-fifth mentioned other antimalarials (chloroquine13.2 % and quinine 9.4 %), about a quarter (22.9 %) stated medicines not belonging to the antimalarial class such as antipyretics/analgesics, and the rest (46.4 %) said they did not know.

#### Knowledge about national policies and guidelines

Only one-third (34.4 %) of caregivers reported knowing that the recommended first-line treatment was ACT, but no one was aware that ACT was not recommended for children younger than four months of age. However, a higher proportion (70.5 %) of caregivers were aware to start an antimalarial within 24-h of the child experiencing fever, with most (93.4 %) reporting that this expectation was attainable.

#### Knowledge about where to access the best antimalarials and advice about malaria

About three-quarters (75.5 %) of caregivers believed that the best medicines were available from public health facilities, pharmacies, private clinics, and CHWs. A similar portion (74.8 %) reported ACTs should be available for free at public health facilities and from CHWs, but only 32.8 % said they would be able to get an ACT from public sources if they needed it for their child. A comparably high percentage (78.3 %) of caregivers identified trained health providers such as doctors, nurses, and CHWs to be the best individuals from whom to obtain advice about antimalarials.

### Assistance with critical decisions

This section evaluated caregivers’ expressed preference with seeking assistance, when deciding about an antimalarial, compared to what occurred for the index child.

A large majority of caregivers (78.3 %) indicated that - given the choice - they would seek advice from a trained health professional on how to treat malaria for children five and under. However, what actually happened for the index child fell slightly short of what caregivers indicated as their preference.

On average, the 290 index children received 1.3 antimalarials over the course of their illness. Of these, 194 children received only one antimalarial, 82 received two antimalarials, and 14 received three antimalarials. For the first antimalarial given, fewer than three-quarters (66.2 %) of the 290 caregivers reported starting this at the advice of trained health professionals, 21.2 % were started at the advice of drug shop vendors, and 7.2 % said they made the decision themselves. However, decisions to give an additional antimalarial commonly occurred at the advice of health providers. A majority (85.4 %) of caregivers reported giving a second antimalarial at the advice of trained health professionals, with few relying on drug shop vendors (6.1 %) or themselves (4.9 %). Similarly, of those caregivers who gave a third or fourth antimalarial, a large portion indicated these decisions had been made by trained health professionals (78.6 % and 100 %, respectively), with only 14.2 % of caregivers and 7.1 % of drug shop vendors deciding on the third antimalarial. On the day of the survey, only 15 of the 390 antimalarials were still being given. Of the antimalarials stopped, two-fifths (44.8 %) had been stopped at the instruction of trained health professionals, caregivers themselves made this decision in the other two-fifth (39.4 %) of the cases, and only rarely were drug shop vendors (9.6 %), family members (3.7 %) or CHWs (0.3 %) involved in this decision.

### Accessing information sources

Information sources accessed by caregivers to learn about approved malaria treatments were grouped into one of the following three information source categories: (1) primary sources - defined as those sources that are most likely to provide up-to-date factual information consistent with national guidelines and are amenable to regular updates. Examples include: public health providers (such as sensitization programs), informed community members (such as CHWs and chairpersons), and news media (such as newspaper, television, and radio), (2) secondary sources - defined as those sources whose factual information can get outdated and are less amenable to regular updates, (3) hearsay – including either (a) information received from family or community members who are not trained health providers, or (b) information learned on their own with no ability to name any sources external to themselves.

On average, for the district, one-third (30.0 %) of caregivers reported they had learned that the “national policy recommended ACT as the first-line treatment” from a primary source, 69.1 % reported they learned this information from hearsay (of which 67.7 % said “self”), and 0.9 % reported a secondary source. While almost all caregivers (93.4 %) reported knowing “when best to start an antimalarial after noticing fever”, only about half (55.2 %) mentioned learning this from a primary source, with 44.8 % learning this from hearsay (of which 41.0 % mentioned “self” or said nothing). Similarly, two-thirds (59.4 %) of caregivers reported learning about “where ACT can be obtained from within their community” from a primary source, with two-fifths (40.6 %) reporting hearing about this from hearsay (of which 31.4 % mentioned “self” or said nothing).

### Predicting receipt of an *appropriate* antimalarial treatment

As a first step, bivariate analyses identified nine survey items that differentiated significantly whether a child had or had not received an *appropriate* antimalarial at or better than *p* < .05. Table 4 presents the nine predictors to receiving an *appropriate* antimalarial, plus a few additional items accounting for unusual fractions of variance (η^2^ ≥ 2 %) or possessing other strong conceptual reasons for further testing. Children were most likely to receive an *appropriate* antimalarial if the caregiver reported: being aware that ACT was the nationally recommended antimalarial (*p* = 0.002), that ACT cured the best (*p* < .000), that they would choose ACT if given the choice (*p* < .000), or they kept ACT in the home for future use (*p* < 0.000). Children were also more likely to receive an *appropriate* antimalarial if the child was seen by a health professional (*p* < 0.000), if the child was seen at a public health facility (*p* < 0.000), or if antimalarials were obtained from public health facilities, CHWs or private hospitals/clinics (*p* < 0.000). Other indicators included caregivers’ gender (*p* = 0.042) and child’s age (*p* = 0.015). Female caregivers were more likely to obtain an *appropriate* antimalarial for their child than were male caregivers, (33.1 % versus 20.3 %, *p* = .042), and infants four months and younger were distinctly disadvantaged compared to older children who were more likely to receive an *appropriate* antimalarial (7.1 % versus 34.6 %; *X*^2^ (7, 432) = 13.10, *p* = .000). No other demographic characteristics significantly differentiated between whether a child had received an *appropriate* antimalarial or not.

**Table 4:** Predictors to whether a child received an *appropriate* antimalarial treatment

Survey questions^a^	Total respondents No.	Bivariate analysis				Multivariate analysis (binary logistics step-wise regression)					
		df	*χ*2	*p*-value	ɳ^2^	Step #	Wald (*X* ^2^)	*p*-value	OR	95 % CI	% Accuracy of prediction
*Receipt of an appropriate antimalarial in Butaleja (N = 424): 32 %*											
Community Factors											
Is it easy to reach PHF	414	1	0.00	.969	0.000	--	--	NS	--	--	--
Child’s Personal Factors											
Age (≤4 months vs Older^b^)	423	7	17.34	.015	0.041	4	6.19	.013	5.67	1.44-22.23	83.0
Gender (Male vs Female)	423	1	0.34	.559	0.001	--	--	NS	--	--	--
Birth order	423	2	2.75	.253	0.007	--	--	NS	--	--	--
CGs' Knowledge About AMs											
Awareness gov’t policy recommends (ACT vs Other)	423	1	9.59	.002	0.023	--	--	NS	--	--	--
Which AMs cure the best (ACT vs Other)	423	1	17.55	<.000	0.042	--	--	NS	--	--	--
Given the choice, which AM would you select first (ACT vs Other)	423	1	34.60	<.000	0.082	3	8.35	.004	2.31	1.31-4.07	81.4
Knowing when to start an AM after noticing fever (Within 24 h vs Longer^b^)	423	2	0.36	.834	0.001	--	--	NS	--	--	--
Is CG knowledgeable about ACT being available in the community (Y/N)	416	1	0.06	.805	0.000	--	--	NS	--	--	--
Child’s Disease Presentation											
Was child seen by a health professional (Y/N)	421	1	12.82	<.000	0.030	--	--	NS	--	--	--
Where was the child seen by the health professional (PHF vs Other)	423	1	21.45	<.000	0.051	--	--	NS	--	--	--
Practice/Management Factors											
Was medicine used for subsequent action (Y/N)	423	1	1.19	.275	0.003	--	--	NS	--	--	--
Where was AM (normally) obtained? (Regulated Outlets^c^ vs Other)	423	1	24.25	<.000	0.057	1	71.94	<.000	14.99	8.02-28.02	71.8
Were medicines kept in home over last 6 months for future use (Y/N)	416	1	2.74	.098	0.007	--	--	NS	--	--	--
Types of medicines kept as home remedy for future use on day of survey (ACT vs Other)	423	1	84.92	<.000	0.201	2	44.75	<.000	6.36	3.70-10.93	81.4
CGs’ Personal & Demographic Factors											
Gender (Male vs Female)	419	1	4.12	.042	0.010	--	--	NS	--	--	--
Number of children ≤ 5 years (Only 1 vs More^b^)	423	5	2.81	.729	0.007	--	--	NS	--	--	--

^a^ *Abbreviations*: antimalarial (*AM*), artemisinin combination therapy (*ACT*), caregiver (*CG*), government (*Gov’t*), public health facility (*PHF*), yes/no (*Y/N*)

^b^For logistic regression recoded into two categories

^c^Regulated outlets: community health worker, *PHF*, regulated private outlets

Additionally, a binary logistic regression model determined items that remained associated with the likelihood of receiving an *appropriate* antimalarial when adjusted for other items. The analysis indicated the overall model to be statistically significant (*X*^2^ (4) = 173.80, *p* < .000), with an 83.0 % predictive value. The results of the logistic model are shown in Table 4. Four survey items remained independent predictors of a child receiving an *appropriate* antimalarial. Caregivers possessing these predictors were characterized as knowledgeable and pro-active. In descending order of odds ratio (OR), these items included: (1) caregivers who obtained antimalarials from public health facilities, CHWs, or private hospitals/clinics (OR = 14.99, *p* < .000), (2) caregivers who kept ACT in the home for future use (OR = 6.36, *p* < .000), (3) child’s age older than four months (OR = 5.67, *p* < .013), and (4) caregivers who reported that given the choice, they would select ACT over other antimalarials (OR = 2.31, *p* < .000).

### Regional differences and receiving an *appropriate* antimalarial treatment

Across the entire Butaleja District, 31.6 % of children received an *appropriate* antimalarial, but sharp differences existed among the 10 sub-counties and two town-councils – ranging from 8 % in Mazimasa in the rural north-east to 52 % in Busolwe Town-Council in the more centralized sector of the district (Additional file 7: Figure S6). At either extreme, the rate of receiving an *appropriate* antimalarial falls far short of the government’s own policy of treatment availability, i.e., readily available and free of charge in all communities.

## Discussion

This study is the first to quantify caregivers’ treatment-seeking patterns for presumed malaria in children five and under for Butaleja District. This study is also unique in quantifying the use of *appropriate* antimalarial treatment. Overall, our study established that a substantial gap remains between Uganda’s national malaria policy and caregivers’ malaria management practices in Butaleja. Only one in five children with fever received any blood test whatsoever, two in five received an ACT (alone or in combination with other antimalarials), and even fewer children received an *appropriate* antimalarial treatment.

While a large majority of caregivers reported initiating some form of action within 24-h of noticing symptoms, for three out of four children this represented home management. In Butaleja the use of home management as initial care was more common than what has been reported nationally (72.6 % vs 42-61 %) or in other regions of eastern Uganda (24 %) [15, 20, 21]. Similarly, the proportion of caregivers who used traditional herbs was also higher in Butaleja than elsewhere in eastern Uganda (26.4 % vs 19 %) [22]. Such practices are particularly disconcerting given the findings by Rutebemberwa et al., indicating the use of home management generally, and the use of traditional herbs specifically, to be associated with delayed treatment-seeking at external sources [22].

Over the course of the index child’s febrile illness, about half were reported to have been seen at a public health facility, a proportion larger than what has been previously reported for eastern Uganda (range: 17–26 %) or nationally (21–24 %) [15, 22–25]. Despite this increased rate of visits to public facilities, ACT usage across Butaleja was reported to be 41.0 % and receipt of an *appropriate* antimalarial only 31.6 %. While treatment policies vary across SSA, according to the Uganda malaria treatment guidelines, ACT constituted the first-line antimalarial for 90 % of index children in this study. ACT usage reported in our study was considerably higher than what has been estimated nationally prior to 2011 (21–23 %), but comparable to 2011 and 2012 national estimates (44 %) [15, 20, 26, 27]. None of the Ugandan studies explicitly report on the prevalence of children receiving only the first-line treatments, and it was not possible to determine from these studies the percentage of children for whom ACT was the only antimalarial given. In this regard, our study provides valuable insight by illustrating that an increase in ACT usage does not necessarily translate into receipt of an *appropriate* antimalarial treatment.

The emphasis on ACT usage in other studies is understandable given that prompt treatment with ACT is crucial for preventing malaria deaths in young children. However, relying on ACT usage as the only indicator of success evades much needed policy discussions around the use of multiple antimalarials to manage malaria in young children. Our study found the practice of polypharmacy to be common, with one in three caregivers in Butaleja reporting using multiple antimalarials to manage malaria in their children, often in combination with non-antimalarials. This practice was confirmed in a follow-up study conducted in Butaleja, and has been reported in other regions of Uganda [15, 28–30]. The work by Rutebemberwa et al. found a large majority of caregivers in Iganga District preferred combination therapy, but caregivers’ views about which antimalarials to start a child on varied considerably [28]. Polypharmacy raises several concerns, some of which include increased risk of adverse drug reactions, drug-drug/disease interactions, emergence of resistive strains, unnecessary therapeutic intensity, and drug non-adherence [31].

A further issue of concern with polypharmacy is the unnecessary expenditure incurred by households due to the sale of redundant drugs. In their study, Nabyonga-Orem et al. established that 82 % of caregivers living in mid-eastern districts, which included Butaleja, incurred out-of-pocket expenditures when seeking advice or treatment for the management of malaria in young children, with 70 % incurring expenditures linked to acquisition of medicines [7]. For this region, expenditures related to purchase of medications was 16 % higher than the national average. While the cost due to drug mis-adventuring has not been estimated for children in Uganda, studies in the west estimate such cost to be in the millions of dollars annually [32]. Future studies, therefore, need to take a broader approach when evaluating use of antimalarials by also considering the prevalence of *appropriate* antimalarial treatment.

This study examined caregivers’ responses to 160 different questions representing a variety of treatment-seeking behaviors, to identify those that might distinguish whether a child received an *appropriate* antimalarial. While nine of these individual questions distinguished significantly using bivariate analysis, our logistic regression model identified four independent predictors of receiving an *appropriate* antimalarial: (1) caregivers who obtained antimalarials from public health facilities, CHWs or private hospitals/clinics, (2) caregivers who kept ACT in the home for future use, (3) caregivers who reported that given the choice, they would select ACT over other antimalarials, and (4) child’s age older than four months. Collectively, the logistic model offered a high predictive value, with 83 % of the cases categorized correctly. While others have found an association between visits to public health facilities and receipt of a first-line antimalarial treatment, our study found visits to a public health facility to be an independent predictor only when an antimalarial was dispensed [23, 33]. Additionally, ours is the only study to identify caregivers’ preference for ACT and their practice of keeping home-stock supplies of ACT to be independent predictors of receiving an *appropriate* antimalarial. With respect to demographic characteristics, only the index child’s age was found to be an independent predictor. The lack of association with other demographic characteristics was not surprising, considering that caregivers in Butaleja were mostly peasant farmers, thus their range of differences for educational and socioeconomic variables may not have been large. National studies in Uganda have also found treatment for fever to vary with a child’s age, with fewer children under the age of one reported to have received an antimalarial or an antimalarial within the same or next day compared to one to five year olds [15, 20].

In rural communities such as Butaleja, where licensed private outlets are few and far between, and most households are too poor to visit private clinics, public health facilities remain the only source for ACTs. Within this context, it is not surprising that obtaining an antimalarial from a public health facility was found to be a significant predictor of receiving an *appropriate* antimalarial. What is noteworthy in this study is that fewer than half of the caregivers reported visiting a public health facility to manage their child’s febrile illness. Of those who did visit a public facility, only 63.0 % of children received an ACT. Given that the use of home-stock medicines and visits to an unlicensed vendor remain common practice, only 43.2 % of those who visited a public health facility could be classified as having received an *appropriate* antimalarial. These findings confirm what has previously been reported for management of childhood malaria in Uganda: the use of public health facilities is limited, visits to a public facility does not ensure receipt of an ACT, and self-management practices with sub-optimal treatments at the household level remains a major obstacle to a child receiving an *appropriate* antimalarial treatment [34]. Accordingly, this study proposes a need for public health education to influence caregivers’ malaria management practices by educating them about ACTS, the importance of using only the first-line treatments, and to know where to quickly obtain these treatments when a child experiences fever. However, in the context of a weak public health system and limited access to licensed private outlets, such initiatives alone will have negligible impact. These findings suggest that limiting ACT access to only public health facilities and licensed private outlets in rural settings such as Butaleja is unlikely to improve malaria management to acceptable levels. To improve access to ACTs across the entire population spectrum, future public health initiatives in Uganda will need to consider strengthening the health system as a whole - including making training programs and subsidized ACTs more widely available at licensed and unlicensed private providers.

### Limitations

The findings from this study need to be considered in the context of potential limitations. First, the study relied on caregivers’ self-reported information. We, therefore, recognize the potential for recall and reporting biases to have influenced the quality of data collected. However, since caregivers were asked to focus on their youngest child who became sick in the previous two weeks of the survey, and the child was present during the interview to help trigger details about the febrile episode, we believe that recall bias was minimized. Similarly, reporting bias was minimized by assuring caregivers confidentiality and privacy during the interview. Furthermore, misclassification of treatments given to index children was reduced by confirming all caregivers’ verbal responses against laminated posters displaying photographs of medicines commonly used for malaria in Butaleja. Lastly, recruitment of the index child was based on presumptive diagnosis rather than on confirmed diagnosis of malaria, which could have resulted in selection of children who may be suffering from diseases other than malaria. Since fever commonly serves as a proxy for malaria in regions such as Butaleja where rapid or microscopic blood testing is rare, our selection process followed real life practices. However, given that this study was conducted during a period of high malaria transmission, the sensitivity of fever being malaria was likely high.

## Conclusions

Uganda’s 2010 and 2015 targets of ensuring 85 % of children under five with fever receive malaria management according to national guidelines are far from being met. In Butaleja, receipt of an *appropriate* antimalarial treatment in young children is undermined by limited access to ACTs during acute episodes of malaria, over-reliance of households on home management, and a lack of caregiver knowledge of correct malaria treatment. Future public health policies and programs, therefore, need to address the shortages of essential malaria commodities in recommended and frequently accessed treatment locations. Particular emphasis should be placed on integrating the unlicensed private sector into standardized care, and on educating caregivers on the importance of confirmatory testing before proceeding to treatment, and on where free ACTs may be accessed within the community.

## Additional files

Additional file 1: Figure S1. Map of Butaleja District. (DOCX 93 kb) Additional file 2: Survey, Survey used to collect information about caregivers’ malaria related treatment-seeking behaviors in Butaleja District. (PDF 1230 kb) Additional file 3: Figure S2. Types and frequency of home management use: alone or in combination (*n* = 424). Abbreviations: management (Mgmt); medicines (Meds). (DOCX 24 kb) Additional file 4: Figure S3. Select first actions taken by caregivers (*n* = 424). Abbreviations: artemisinin combination therapy (ACT); antimalarial (AM); malaria (MA); management (Mgmt); trained health professional (HP); within (W/I). (DOCX 64 kb) Additional file 5: Figure S4. Select actions taken by caregivers over the course of the child’s fever episode (*n* = 424). Abbreviations: antimalarial (AM); public health facility (PHF); within (W/I). (DOCX 52 kb) Additional file 6: Figure S5. Antimalarial use over the course of the child’s fever episode (*n* = 424). Abbreviations: appropriate (Apprp); artemisinin combination therapy (ACT); antimalarial (AM). (DOCX 24 kb) Additional file 7: Figure S6. Probability of receiving an appropriate antimalarial treatment across different regions of Butaleja District. (DOCX 69 kb)

## Abbreviations

ACT: Artemisinin combination therapy
AM: Antimalarial
AMFm: Affordable Medicines Facility-malaria
Apprp: Appropriate
CG: Caregiver
CI: Confidence Interval
CIHR: Canadian Institute of Health Research
Df: Degrees of freedom
Gov’t: Government
HP: trained health professional
iCCM: Integrated community case management
MA: Malaria
Meds: Medicines
Mgmt: Management
OR: Odds Ratio
PHF: Public health facility
SSA: Sub-Saharan Africa
W/I: Within
WHO: World Health Organization
Y/N: Yes/no
η2: eta-squared
*χ*2: chi-square test

## References

[1] United Nations Children's Fund. Invest in the future: defeat malaria. World malaria day 2013: focus on Africa. 2013. http://www.unicef.org/health/files/Malaria_brochure_18April2013.pdf. Accessed 30 Jan 2016.

[2] World Health Organization. Malaria: malaria in infants. 2013. http://www.who.int/malaria/areas/high_risk_groups/infants/en/. Accessed 30 Jan 2016.

[3] World Health Organization. World malaria report 2012: fact sheet. http://www.who.int/malaria/publications/world_malaria_report_2012/wmr2012_factsheet.pdf. Accessed 30 Jan 2016.

[4] Murray CJ, Rosenfeld LC, Lim SS, Andrews KG, Foreman KJ, Haring D (2012). Global malaria mortality between 1980 and 2010: a systematic analysis. Lancet.

[5] Yeka A, Gasasira A, Mpimbaza A, Achan J, Nankabirwa J, Nsobya S (2012). Malaria in Uganda: challenges to control on the long road to elimination: I. Epidemiology and current control efforts. Acta Trop.

[6] Okello PE, Van Bortel W, Byaruhanga AM, Correwyn A, Roelants P, Talisuna A (2006). Variation in malaria transmission intensity in seven sites throughout Uganda. Am J Trop Med Hyg.

[7] Nabyonga-Orem J, Mugisha F, Okui AP, Musango L, Kirigia JM (2013). Health care seeking patterns and determinants of out-of-pocket expenditure for malaria for the children under-five in Uganda. Malar J.

[8] Ministry of Health. Uganda malaria control strategic plan: 2005/06 - 2009/10. 2005. http://archiverbm.rollbackmalaria.org/countryaction/nsp/uganda.pdf. Accessed 30 Jan 2016.

[9] Ministry of Health Uganda. Uganda Clinical Guideline 2010: National Guidelines for Management of Common Conditions. Kampala: The Republic of Uganda; 2010.

[10] Kassam R, Collins JB, Liow E, Rasool N (2015). Narrative review of current context of malaria and management strategies in Uganda (Part I). Acta Trop.

[11] President's Malaria Initiative. President's Malaria Initiative Uganda malaria operational plan for FY 2015. 2015. https://www.pmi.gov/docs/default-source/default-document-library/malaria-operational-plans/fy-15/fy-2015-uganda-malaria-operational-plan.pdf?sfvrsn=3. Accessed 03 Sept 2016.

[12] Uganda (2009). Higher Local Government Statistical Abstract.

[13] Wikipedia. Butaleja District. http://en.wikipedia.org/wiki/Butaleja_District. Accessed 30 Jan 2016.

[14] Butaleja District Local Government NEMAN, United Nations Development Programme (UNDP)/Poverty and Environment Initiatives (PEI). District Environmental Policy - Butaleja. 2009. http://www.nemaug.org/district_policies/Butaleja_District_Environment_Policy.pdf. Accessed 30 Jan 2016.

[15] ACTwatch Group. Household survey, Uganda, 2012 survey report. 2013. http://www.actwatch.info/sites/default/files/content/publications/attachments/ACTwatch%20HH%20Report%20Uganda%202012.pdf. Accessed 30 Jan 2016.

[16] Nanyunja M, Nabyonga-Orem J, Kato F, Kaggwa M, Katureebe C, Saweka J (2011). Malaria treatment policy change and implementation: the case of Uganda. Malar Res Treat.

[17] Raosoft, Inc. Sample size calculator. http://www.raosoft.com/samplesize.html. Accessed 30 Jan 2016.

[18] Fink G, Dickens WT, Jordan M, Cohen JL (2014). Access to subsidized ACT and malaria treatment--evidence from the first year of the AMFm program in six districts in Uganda. Health Policy Plan.

[19] Bartoloni A, Zammarchi L (2012). Clinical aspects of uncomplicated and severe malaria. Mediterr J Hematol Infect Dis.

[20] ACTwatch Group. Household survey report (baseline) Republic of Uganda 03/09-04/09. 2009. http://www.actwatch.info/sites/default/files/content/publications/attachments/Uganda%20Household%20Baseline%2C%20ACTwatch%202009.pdf. Accessed 30 Jan 2016.

[21] Awor P, Wamani H, Bwire G, Jagoe G, Peterson S (2012). Private sector drug shops in integrated community case management of malaria, pneumonia, and diarrhea in children in Uganda. Am J Trop Med Hyg.

[22] Rutebemberwa E, Kadobera D, Katureebe S, Kalyango JN, Mworozi E, Pariyo G (2012). Use of community health workers for management of malaria and pneumonia in urban and rural areas in eastern Uganda. Am J Trop Med Hyg.

[23] Littrell M, Gatakaa H, Evance I, Poyer S, Njogu J, Solomon T (2011). Monitoring fever treatment behaviour and equitable access to effective medicines in the context of initiatives to improve ACT access: baseline results and implications for programming in six African countries. Malar J.

[24] Rutebemberwa E, Kallander K, Tomson G, Peterson S, Pariyo G (2009). Determinants of delay in care-seeking for febrile children in eastern Uganda. Trop Med Int Health.

[25] Rutebemberwa E, Pariyo G, Peterson S, Tomson G, Kallander K (2009). Utilization of public or private health care providers by febrile children after user fee removal in Uganda. Malar J.

[26] Uganda Bureau of Statistics (UBOS). Uganda demographic and health survey 2011. 2012. http://www.ubos.org/onlinefiles/uploads/ubos/UDHS/UDHS2011.pdf. Accessed 30 Jan 2016.

[27] Uganda Bureau of Statistics (UBOS), Uganda Malaria Surveillance Project Molecular Laboratory, National Malaria Control Programme, ICF Macro. Uganda malaria indicator survey 2009. 2010;1-144. http://www.measuredhs.com/pubs/pdf/MIS6/MIS6.pdf. Accessed 30 Jan 2016.

[28] Rutebemberwa E, Nsabagasani X, Pariyo G, Tomson G, Peterson S, Kallander K (2009). Use of drugs, perceived drug efficacy and preferred providers for febrile children: implications for home management of fever. Malar J.

[29] Tabuti JR (2008). Herbal medicines used in the treatment of malaria in Budiope county. Uganda J Ethnopharmacol.

[30] Kassam R, Sekiwunga R, MacLeod D, Tembe J, Liow E (2016). Patterns of treatment-seeking behaviors among caregivers of febrile young children: a Ugandan multiple case study. BMC Public Health.

[31] Manasse HR (1995). Toward defining and applying a higher standard of quality for medication use in the United States. Am J Health Syst Pharm.

[32] Johnson JA, Bootman JL (1995). Drug-related morbidity and mortality. A cost-of-illness model. Arch Intern Med.

[33] Kemble SK, Davis JC, Nalugwa T, Njama-Meya D, Hopkins H, Dorsey G, Staedke SG (2006). Prevention and treatment strategies used for the community management of childhood fever in Kampala. Uganda Am J Trop Med Hyg.

[34] Kassam R, Collins JB, Liow E, Rasool N (2015). Caregivers’ treatment-seeking behaviors and practices in Uganda - a systematic review (Part II). Acta Trop.

