# Supplementary figures and images for: Caregivers’ treatment-seeking behaviors and predictors of whether a child received an appropriate antimalarial treatment: a household survey in rural Uganda
Source: BMC Infect Dis. 2016 Sep 6;16(1):478. doi: 10.1186/s12879-016-1815-5 (PMC5012054; doi:10.1186/s12879-016-1815-5)

**
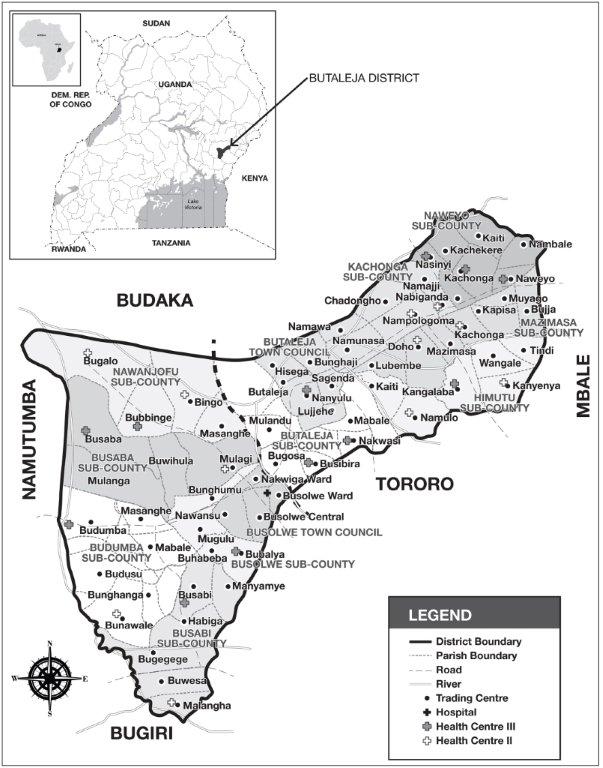
**

Supplement: Additional file 1: Figure S1. — Map of Butaleja District. (DOCX 93 kb) [file 12879_2016_1815_MOESM1_ESM.docx]

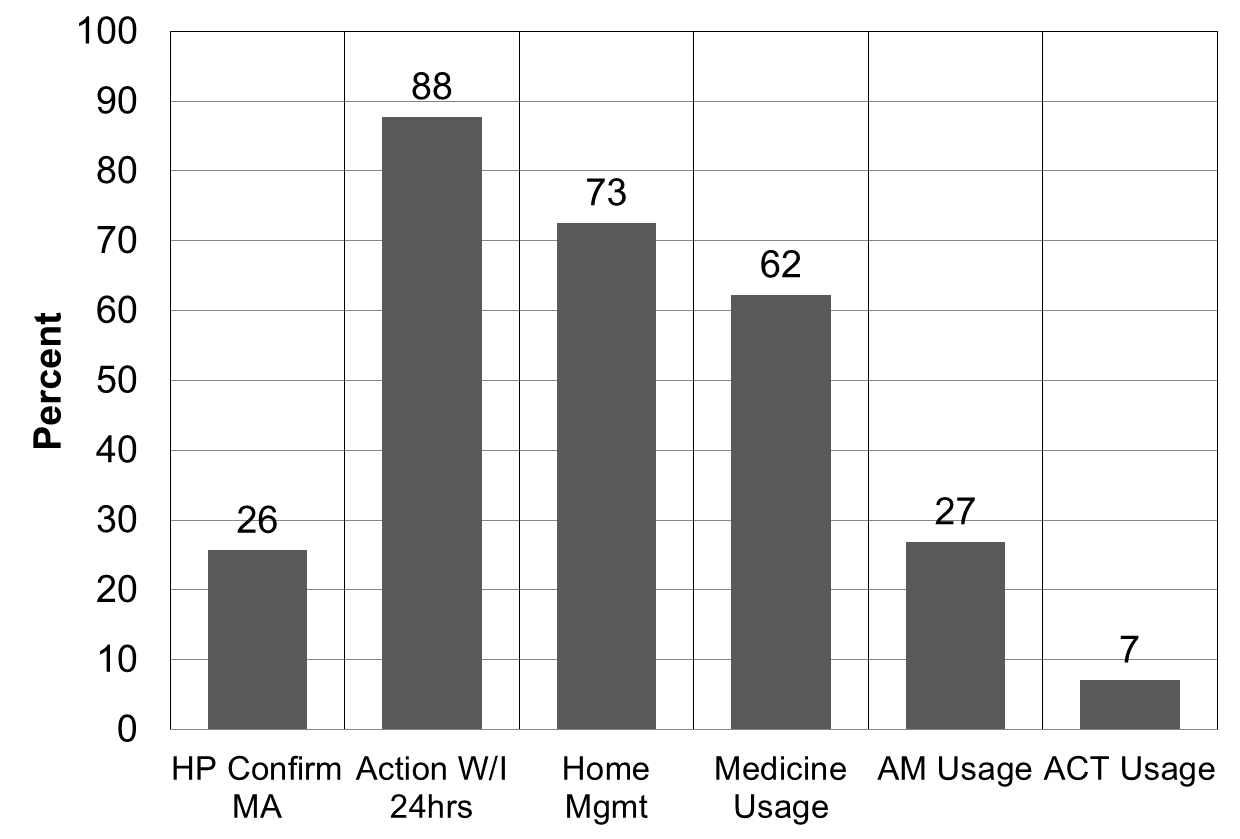

Supplement: Additional file 4: Figure S3. — Select first actions taken by caregivers (n = 424). Abbreviations: artemisinin combination therapy (ACT); antimalarial (AM); malaria (MA); management (Mgmt); trained health professional (HP); within (W/I). (DOCX 64 kb) [file 12879_2016_1815_MOESM4_ESM.docx]

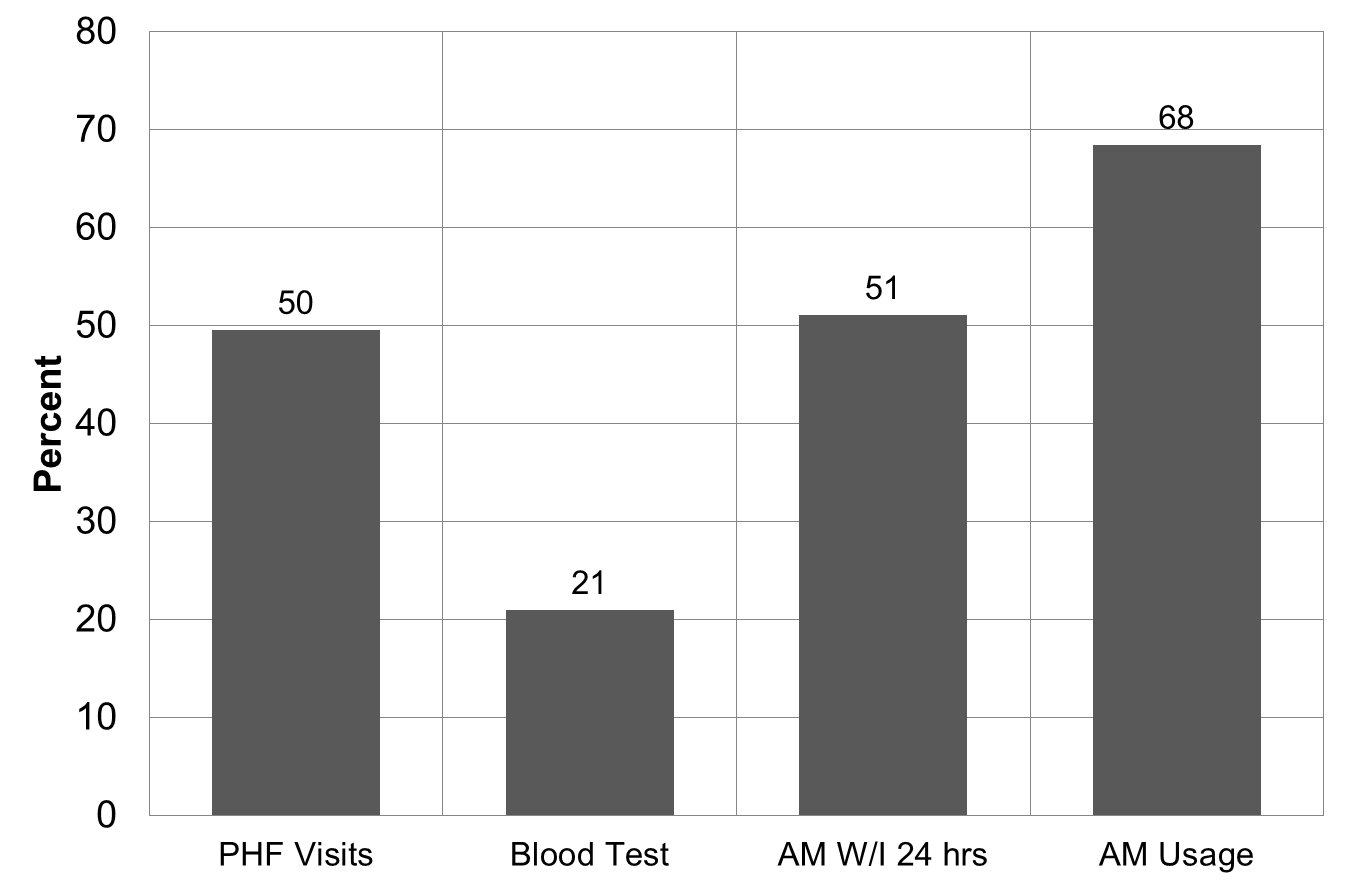

Supplement: Additional file 5: Figure S4. — Select actions taken by caregivers over the course of the child’s fever episode (n = 424). Abbreviations: antimalarial (AM); public health facility (PHF); within (W/I). (DOCX 52 kb) [file 12879_2016_1815_MOESM5_ESM.docx]

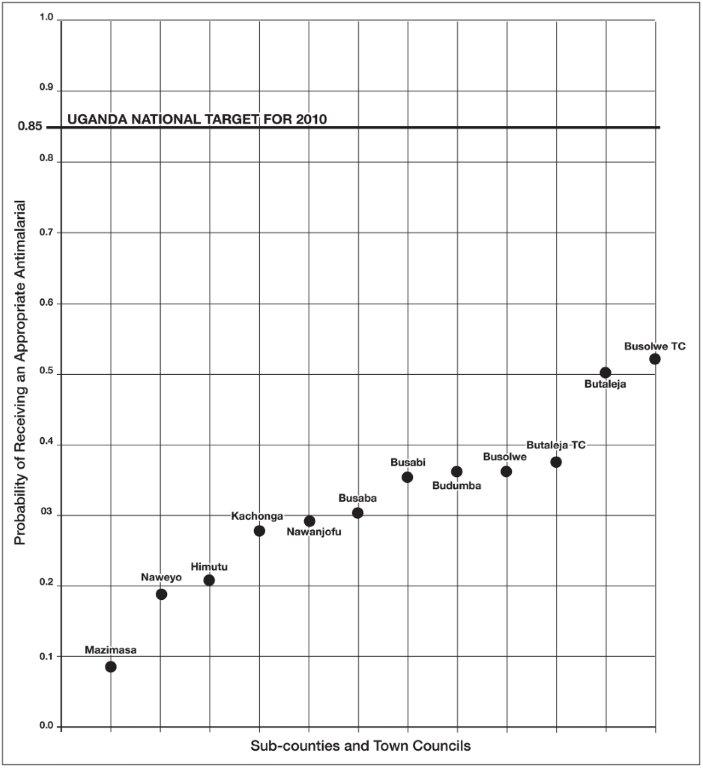

Supplement: Additional file 7: Figure S6. — Probability of receiving an appropriate antimalarial treatment across different regions of Butaleja District. (DOCX 69 kb) [file 12879_2016_1815_MOESM7_ESM.docx]
