# Supplementary material for: Caregivers’ treatment-seeking behaviors and predictors of whether a child received an appropriate antimalarial treatment: a household survey in rural Uganda
Source: BMC Infect Dis. 2016 Sep 6;16(1):478. doi: 10.1186/s12879-016-1815-5 (PMC5012054; doi:10.1186/s12879-016-1815-5)
Supplement: Additional file 2: — Survey, Survey used to collect information about caregivers’ malaria related treatment-seeking behaviors in Butaleja District. (PDF 1230 kb) [file 12879_2016_1815_MOESM2_ESM.pdf]

CAREGIVER SURVEY

a) GREET THE PEOPLE YOU WILL FIND AT EACH HOUSEHOLD:
Good morning/afternoon

b) ASK FOR THE CAREGIVER OR RESPONSIBLE ADULT HOUSEHOLD MEMBER

c) INTRODUCE YOURSELF THUS:
I am (name of interviewer), a research assistant with the Butaleja Malaria Project, a partnership between the University of Makerere and Canada.

d) EXPLAIN THE PURPOSE OF YOUR VISIT:
The purpose of my visit is to …

e) ASK THE CAREGIVER OR ADULT HOUSEHOLD MEMBER WHETHER THERE ARE ANY CHILDREN 5 YEARS OF AGE AND UNDER IN THE HOUSEHOLD:

☐ YES CONTINUE DOWN THIS PAGE
☐ NO STOP THE INTERVIEW AND GO TO THE NEXT HOUSEHOLD

f) IF NOT SPEAKING TO THE CAREGIVER, ASK TO SPEAK WITH THE CAREGIVER OF CHILDREN 5 YEARS OF AGE AND UNDER IN THE HOUSEHOLD

g) ASK: Did any of your children 5 years of age and under experience fever in the last 2 weeks?

☐ YES OBTAIN INFORMED CONSENT AND START INTERVIEW
☐ NO STOP THE INTERVIEW, GO TO THE NEXT HOUSEHOLD

INTERVIEWER'S VISIT INFORMATION: DATE: DAY:____MONTH:____YEAR: ____

INTERVIEWER'S NAME:____

RECORD BEGINNING TIME: ____

RESULT OF VISIT: (If caregiver refuses to participate, provide reason)
____
____
____

IDENTIFICATION

HOUSEHOLD NUMBER: _____/_____/_________

HEALTH SUB DISTRICT: 1.______________________ 2.________________________________

BUTALEJA SUB-COUNTY: _________________________ **PARISH:** ____

VILLAGE: ____

CAREGIVER STUDY ID#: ____

IS CAREGIVER PART OF THIS HOUSEHOLD? 1. YES ☐ 0. NO ☐

WHAT IS CAREGIVER'S RELATIONSHIP TO HEAD OF HOUSEHOLD

1. Wife	2. Husband	3. Daughter	4. Son	5. Sister	6. Brother	7. Grandchild	8. Mother
9. Father	10. Son-in-law	11. Daughter-in-law	12. Aunt	13. Uncle	14. Grandparent	15. Household head	

16. Other (Specify): ____

CAREGIVER PART OF THIS HOUSEHOLD? 1. YES ☐ 0. NO ☐

INFORMATION OF ALL CHILDREN 5 YEARS AND UNDER WITH FEVER IN THE LAST 2 WEEKS (STARTING WITH THE YOUNGEST)

	Name	Sex	Birth date Month/Year	Age (years)
Youngest		1.Male; 2.Female		
2nd Youngest		1.Male; 2.Female		
		1.Male; 2.Female		
		1.Male; 2.Female		
		1.Male; 2.Female		
		1.Male; 2.Female		
		1.Male; 2.Female		

SELECT THE YOUNGEST TO BECOME THE INDEX CHILD FOR THIS INTERVIEW

WHAT IS CAREGIVER'S RELATIONSHIP TO INDEX CHILD:

1. Wife	2. Husband	3. Daughter	4. Son	5. Sister	6. Brother	7. Grandchild	8. Mother
9. Father	10. Son-in-law	11. Daughter-in-law	12. Aunt	13. Uncle	14. Grandparent	15. Household head	

16. Other (Specify): ____

PART 1. QUESTIONS FOR ALL CAREGIVERS

(DIRECTIONS TO INTERVIEWER) ASK: "I now want to ask you about this child's illness related to this fever episode."

1.	What first sign(s) and symptom(s) did you see your child have when s/he had the illness we are now talking about? **(CIRCLE ALL THAT APPLY)**				
	1. Feeling cold (Chills/Shivering)<br>2. Body ache<br>3. Chest pains<br>4. Cough<br>5. Diarrhea	6. Fever/high temperature/hot body<br>7. Headache<br>8. Joint /muscle pain<br>9. Malaise<br>10. Abdominal Pain	11. Decreased Urination<br>12. Black Stools<br>13. Unable to sit<br>14. Pale eyes<br>15. Pale palms	16. poor appetite<br>17. Vomiting<br>18. Weakness<br>19. Abnormal/Rapid breathing<br>20. Convulsions	21. Nausea<br>22. Jaundice (Yellow colour)<br>23. Unable to eat/drink/ breastfeed<br>24. Unconsciousness (coma)<br>96. Other (specify)________________
2.	What time of the day were the first sign(s) and symptom(s) first noticed?				
	1. Morning<br>2. Afternoon	3. Dusk/early evening<br>4. Night time	5. Midnight to dawn		
3.	Which individual assisted you in recognizing this child was ill?				
	**HEALTH PROFESSIONAL:**<br>1. Doctor<br>2. Pharmacist<br>3. Nurse<br>96. Other (specify) ________________	**OTHER (RELATION TO CAREGIVER):**<br>4. Spouse<br>5. Father<br>6. Mother	7. Grandmother<br>8. In-Law<br>9. Sibling<br>10. Neighbour	11. Drug shop personnel<br>12. Traditional healer<br>13. No one else (Myself)<br>97. Other (specify) ______________________	
4.	Is this child still ill from this fever episode?				
	1. Yes	0. No			
5.	What illness was (is) this child suffering from?				
	1. Malaria<br>2. Diarrhea<br>3. Tuberculosis	4. Enlarged spleen<br>5. Fever<br>6. Measles	7. Pneumonia<br>8. Stomach wounds<br>90. Don't know	96. Other (specify)____________________________	
6.	Which individual assisted you in recognizing that this child was suffering from this illness?				
	**HEALTH PROFESSIONAL:**<br>1. Doctor<br>2. Pharmacist<br>3. Nurse<br>96. Other (specify) ________________	**OTHER (RELATION TO CAREGIVER):**<br>4. Spouse<br>5. Father<br>6. Mother	7. Grandmother<br>8. In-Law<br>9. Sibling<br>10. Neighbour	11. Drug shop personnel<br>12. Traditional healer<br>13. No one else (Myself)<br>97. Other (specify) ______________________	
	**(DIRECTIONS TO INTERVIEWER):**<br>**IF MALARIA WAS *NOT SUSPECTED* and THIS WAS DIAGNOSED BY HEALTH PROFESSIONAL, THEN STOP THE INTERVIEW FOR *THIS* CHILD.**<br>**ASK QUESTIONS 1 TO 6 FOR THE *NEXT YOUNGEST* CHILD 5 YEARS OF AGE AND UNDER WITH FEVER IN THE PAST 2 WEEKS.**<br>**IF *NO CHILDREN* HAVE FEVER OR WERE DIAGNOSED AS NOT HAVING MALARIA BY HEALTH PROFESSIONAL, THEN STOP INTERVIEW AT THIS HOUSEHOLD.**<br>Thank caregiver for their time and answers; let them know this information will still be useful in this study.				

7.	Can you tell me the one main sign or symptom of malaria in a child 5 years and under?				
	1. Feeling cold (Chills/Shivering)<br>2. Body ache<br>3. Chest pains<br>4. Cough<br>5. Diarrhea	6. Fever/high temperature/hot body<br>7. Headache<br>8. Joint /muscle pain<br>9. Malaise<br>10. Abdominal Pain	11. Decreased Urination<br>12. Black Stools<br>13. Unable to sit<br>14. Pale eyes<br>15. Pale palms	16. poor appetite<br>17. Vomiting<br>18. Weakness<br>19. Abnormal/Rapid breathing<br>20. Convulsions	21. Nausea<br>22. Jaundice (Yellow colour)<br>23. Unable to eat/drink/ breastfeed<br>24. Unconsciousness (coma)<br>90. Don't know<br>96. Other (specify) ________________
8.	Can you tell me other signs and symptoms of malaria in a child 5 years and under? **(CIRCLE ALL THAT APPLY)**				
	1. Feeling cold (Chills/Shivering)<br>2. Body ache<br>3. Chest pains<br>4. Cough<br>5. Diarrhea	6. Fever/high temperature/hot body<br>7. Headache<br>8. Joint /muscle pain<br>9. Malaise<br>10. Abdominal Pain	11. Decreased Urination<br>12. Black Stools<br>13. Unable to sit<br>14. Pale eyes<br>15. Pale palms	16. poor appetite<br>17. Vomiting<br>18. Weakness<br>19. Abnormal/Rapid breathing<br>20. Convulsions	21. Nausea<br>22. Jaundice (Yellow colour)<br>23. Unable to eat/drink/ breastfeed<br>24. Unconsciousness (coma)<br>90. Don't Know<br>96. Other specify) _____________
9.	What is the one main cause for malaria?				
	1. Cold or changing weather<br>2. Drinking dirty water<br>3. Eating cold cornmeal	4. Eating dirty food<br>5. Eating immature sugar cane<br>6. Eating bad food	7. Getting soaked with rain<br>8. Mosquito bites<br>9. Witchcraft	10. Birds<br>11. Dirty household surrounding<br>90. Don't know	96. Other (specify)___________
10.	Where have you heard or seen information about malaria?				
	1. Wall painting<br>2. Calendar<br>3. Drug shop provider<br>4. Health care staff	5. Medicine package<br>6. Leaflet (flyer)<br>7. Newspaper<br>8. Posters	9. Radio<br>10. Family members. Specify __________________<br>11. Neighbour	12. Other community members. Specify __________________<br>13. Fellow workers<br>14. Through training course	15. Through barazas<br>16. TV<br>17. No one else (Myself)<br>96. Other (specify) __________________
11.	Was this child seen by a health professional (doctor, nurse or medical attendant)? Other _____________________				
	1. Yes, where ____	0. No			
12.	Was a blood test done to confirm whether or not the child had malaria?				
	1. Yes,<br>**IF YES,** where was the test done ____	0. No			

(DIRECTIONS TO INTERVIEWER): *SELECT ONE OPTION* ONLY (13.1 or 13.2) TO ANSWER BUT NOT BOTH:

OPTION 13.1. **IF THE CHILD *WAS SUSPECTED* TO HAVE MALARIA**

13.1.1 Can you tell me what signs and symptoms alerted you that this child might have malaria? (**CIRCLE ALL THAT APPLY**)

1. Feeling cold (Chills/Shivering)<br>2. Body ache<br>3. Chest pains<br>4. Cough<br>5. Diarrhea	6. Fever/high temperature/hot body<br>7. Headache<br>8. Joint /muscle pain<br>9. Malaise<br>10. Abdominal Pain	11. Decreased Urination<br>12. Black Stools<br>13. Unable to sit<br>14. Pale eyes<br>15. Pale palms	16. poor appetite<br>17. Vomiting<br>18. Weakness<br>19. Abnormal/Rapid breathing<br>20. Convulsions	21. Nausea<br>22. Jaundice (Yellow colour)<br>23. Unable to eat/drink/ breastfeed<br>24. Unconsciousness (coma)<br>90. Don't Know<br>96. Other specify) ______________

13.1.2. In your opinion, was this child's malaria mild or severe?

1. Mild	2. Severe

OPTION 13.2. **IF THE CHILD WAS *NOT SUSPECTED* TO HAVE MALARIA:**

13.2.1. What signs and symptoms alerted you that this child might not have had malaria? **(CIRCLE ALL THAT APPLY)**

1. Feeling cold (Chills/Shivering)<br>2. Body ache<br>3. Chest pains<br>4. Cough<br>5. Diarrhea	6. Fever/high temperature/hot body<br>7. Headache<br>8. Joint /muscle pain<br>9. Malaise<br>10. Abdominal Pain	11. Decreased Urination<br>12. Black Stools<br>13. Unable to sit<br>14. Pale eyes<br>15. Pale palms	16. poor appetite<br>17. Vomiting<br>18. Weakness<br>19. Abnormal/Rapid breathing<br>20. Convulsions	21. Nausea<br>22. Jaundice (Yellow colour)<br>23. Unable to eat/drink/ breastfeed<br>24. Unconsciousness (coma)<br>90. Don't Know<br>96. Other specify) ______________

(DIRECTIONS TO INTERVIEWER) SAY:
"I want to now ask you about the <u>very first action</u> that was taken when this child had this episode of fever we are now talking about:"

14. What form of action did your child receive? **(CIRCLE ALL THAT APPLY)**

1. No action<br>2. Home remedy	3. Traditional remedy<br>4. Prayer	5. Western medicine

IF ACTION INCLUDED TRADITIONAL OR HOME REMEDY, LIST WHAT THEY WERE BELOW:

TRADITIONAL REMEDY	HOME REMEDY

- **IF ACTION INCLUDED WESTERN MEDICINES, LIST THE NAMES OF ALL MEDICINES UNDER <u>Q.26a</u> - IN SPACE PROVIDED FOR *FIRST ACTION***

15. When was the very first action started after fever was noticed?

1. No action was started<br>2. Immediately (within 6 hour)<br>3. Same day (within 1 day or 24 hours)	4. Next day (on the 2nd day or beyond 24 hours)<br>5. More than 2 days later (more than 48 hours)<br>96. Other (specify)_________________

(DIRECTIONS TO INTERVIEWER): REFER TO Q.14 and SELECT ALL OPTIONS (FROM Q.16.1, 16.2, 16.3 and 16.4) THAT APPLY. *CHOOSE* ANSWERS FROM THE *TWO* TABLES PROVIDED BELOW

OPTION 16.1. SELECT IF *NO FORM* OF ACTION or *ONLY PRAYER* WAS PROVIDED:

16.1.1. Why was no form of action provided? REASON FOR A PARTICULAR ACTION (Specify number): ________________________

16.1.2. Who advised you on not starting any form of action?

☐ HEALTH PROFESSIONAL (Specify number): __________________________________ ☐ OTHER RELATION TO CAREGIVER (Specify number): __________________________________

OPTION 16.2. SELECT IF *TRADITIONAL REMEDY* WAS PROVIDED:

16.2.1. Why was traditional remedy provided? REASON FOR A PARTICULAR ACTION (Specify number): __________________________________

16.2.2. Who advised you on starting the traditional remedy?

☐ HEALTH PROFESSIONAL (Specify number): __________________________________ ☐ OTHER RELATION TO CAREGIVER (Specify number): __________________________________

OPTION 16.3. SELECT IF *HOME REMEDY* WAS PROVIDED:

16.3.1. Why was home remedy provided? REASON FOR A PARTICULAR ACTION (Specify number): ________________________

16.3.2. Who advised you on starting the home remedy?

☐ HEALTH PROFESSIONAL (Specify number): __________________________________ ☐ OTHER RELATION TO CAREGIVER (Specify number): __________________________________

OPTION 16.4. SELECT IF *WESTERN MEDICINE* WAS PROVIDED:

16.4.1. Who advised you on starting the western medicine?

☐ HEALTH PROFESSIONAL (Specify number): __________________________________ ☐ OTHER RELATION TO CAREGIVER (Specify number): __________________________________

TABLE: REASONS FOR A PARTICULAR ACTION

1. Fever was not serious, waiting for fever to get worse before seeking treatment	2. Usually first wait and see if fever will go away No money to buy drugs<br>3. No transportation to buy medicine	4. The place to obtain medicine was too far away<br>5. No one in the household had time to obtain treatment	6. Did not know where to go to get treatment<br>7. Medicines not available at drug shop<br>8. Medicines not available at health facility	9. Fever went away<br>10. No medicines at home<br>11. No drug shop open<br>12. No health facility open<br>13. Not recommended by __________________	96. Other (specify): _____________________

TABLE: LIST OF HEALTH PROFESSIONALS and OTHER RELATION TO CAREGIVER

**HEALTH PROFESSIONAL:**	**OTHER (RELATION TO CAREGIVER):**		
1. Doctor<br>2. Pharmacist<br>3. Nurse<br>96. Other (specify) _________________	4. Spouse<br>5. Father<br>6. Mother	7. Grandmother<br>8. In-Law<br>9. Sibling<br>10. Neighbour	11. Drug shop personnel<br>12. Traditional healer<br>13. No one else (Myself)<br>97. Other (specify) _______________________

(DIRECTIONS TO INTERVIEWER): ASK THE FOLLOWING QUESTIONS *WHETHER OR NOT* ANY FORM OF FIRST ACTION WAS TAKEN

17.	If you had a choice, what different form of action would you have selected for this child to treat the current fever episode we are now talking about? **(CHOOSE ALL THAT APPLY)**		
	1. No change, same action<br>2. Traditional remedy	3. Home remedy<br>4. Prayer	5. Western Medicine<br>96. Other (specify) ______________________
18.	Could you have independently decided to start this child on a different form of action or would you have required permission?		
	1. Yes	0. No	

19.	Who makes the final decision on what form of action to start?			
	**HEALTH PROFESSIONAL:**<br>1. Doctor<br>2. Pharmacist<br>3. Nurse<br>96. Other (specify) ____________	**OTHER (RELATION TO CAREGIVER):**<br>4. Spouse<br>5. Father<br>6. Mother	7. Grandmother<br>8. In-Law<br>9. Sibling<br>10. Neighbour	11. Drug shop personnel<br>12. Traditional healer<br>13. No one else (Myself)<br>97. Other (specify) ____________________

20.	Do you believe that the first action for this child would have been different if the child was of the <u>opposite sex</u>? **(READ ANSWER FROM Q.14 TO THE CAREGIVER OF THEIR FIRST ACTION)**	
	1. Yes **,**<br>**IF YES,** How? ________________________________<br>Why? ________________________________	0. No
21.	Do you believe that the first action for this child would have been different if the child was one of your other children 5 years of age and under? **(READ ANSWER FROM Q.14 TO THE CAREGIVER OF THEIR FIRST ACTION)**	
	1. Yes **,**<br>**IF YES,** How? ________________________________<br>Why? ________________________________	0. No
22.	After this first action, was there a need for <u>further action</u> in this child?	
	1. Yes**.**<br>**IF YES, SAY:** I want to ask you what forms of actions were used <u>subsequent to the first action</u>?	0. No,

23.	Were home remedies used?	
	1. Yes<br>**IF YES ASK:** what? ____________________	0. No
24.	Were traditional remedies used?	
	1. Yes<br>**IF YES ASK:** what? ____________________	0. No
25.	Were western medicines used?	
	1. Yes<br>**IF YES, LIST ALL WESTERN MEDICINES UNDER <u>Q.26b</u>**	0. No,<br>**IF NO, ASK:** "What was the main reason for you to not use any western medicines"?<br>(Specify number From table below) ______________

TABLE: REASONS FOR NOT USING WESTERN MEDICINE

1. Fever was not serious, waiting for fever to get worse before seeking treatment	2. Usually first wait and see if fever will go away<br>3. No money to buy drugs<br>4. No transportation to buy medicine	5. The place to obtain medicine was too far away<br>6. No one in the household had time to obtain treatment	7. Did not know where to go to get treatment<br>8. Medicines not available at drug shop<br>9. Medicines not available at health facility	10. Fever went away<br>11. No medicines at home<br>12. No drug shop open<br>13. No health facility open<br>14. Not recommended by ______________	96. Other (specify): ____________

(DIRECTION TO INTERVIEWER) IF NO WESTERN MEDICINES ARE USED IN FIRST AND SUBSEQUENT ACTIONS, THEN GO TO PART 3 (blue pages)

26.	(DIRECTIONS TO INTERVIEWER):<br><br>LIST OF *ALL* WESTERN MEDICINES USED (FROM Q.14 and Q.25)	**26a.**<br>**WESTERN MEDICINES USED IN FIRST ACTION (FROM Q.14)**	**26b.**<br>**WESTERN MEDICINES USED ON SUBSEQUENT ACTION (FROM Q. 25)**
		1.	1.
		2.	2.
		3.	3.
		4.	4.
		5.	5.
		6.	6.

27. **(DIRECTIONS TO INTERVIEWER): DETERMINE IF WESTERN antiMALARIA MEDICINES WERE USED BY COMPARING THE LIST GENERATED ABOVE IN Q.26 & b WITH THE WESTERN antiMALARIA MEDICINE LIST PROVIDED IN THE TABLE BELOW (USE PICTURES AS SUBSTITUTE FOR LIST)**

1. Yes,
   IF YES, LIST THE antiMALARIA WESTERN MEDICINES USED, STARTING WITH FIRST

   1. ____
   2. ____
   3. ____
   4. ____
   5. ____
   6. ____

   GO TO PART 2 (Q. 28).

0. No,
   IF NO, GO TO PART 3 (BLUE PAGES)

TABLE: WESTERN antiMALARIA MEDICINE LIST

Amodiaquine	Artesunate	Clindamycin	Isoniazid	Pyrimethamine	Sulfalene
Arteether	Atovaquone	Dapsone	Lumefantrine	Pyronaridine	Sulfamethoxazole
Arteflene	Cloroquine	Dihydroartemisinin	Mefloquine	Quinidine	Sulfamethoxypyrazine
Artemether	Chlorproguanil	Doxycycline	Naphthoquine	Quinine	Sulfametopyrazine
Artemisinin	Cinchonidine	Halofantrine	Piperaquine	Rifampicin	Tafenoquine
Artemotil	Cinchonine	Hydroxychloroquine	Proguanil	Sulfadoxine	Tetracycline

Other: ____

PART 2. (DIRECTION TO INTERVIEWERS): THIS SECTION IS FOR CAREGIVERS WHOSE CHILDREN *RECEIVED antiMALARIA* WESTERN MEDICINES (REFER TO Q. 27)
Skip to Part 3 Question #92 if did not received a western antimalarial medicine (blue sheets)

INTERVIEWER, SAY: **"**I now want to ask you about each of the **anti**malaria western medicine that was given to this child**".**

**(DIRECTIONS TO INTERVIEWER): LIST HERE ALL WESTERN antiMALARIA MEDICINES FROM Q.27**	28. Who decided this child should be given this **anti**malaria medicine? **(SELECT FROM LIST BELOW)**			29. What was the one main reason for choosing this **anti**malaria medicine? **(CHOOSE FROM LIST BELOW)**
	**HEALTH PROFESSIONAL:**	**OTHER (RELATION TO CAREGIVER):**		1. Already in the home<br>2. Only one available at drug shop<br>3. Cheapest one at drug shop<br>4. Recommended by drug shop provider<br>5. Considered the most effective<br>6. Prescribed by medical practitioner<br>7. Traditional or herbal medicine or prayer was not sufficient<br>96. Other (specify) ____________________
	1. Doctor<br>2. Pharmacist<br>3. Nurse<br>96. Other (specify) ____________	4. Spouse<br>5. Father<br>6. Mother<br>7. Grandmother In-Law<br>8. Sibling	9. Neighbour<br>10. Drug shop personnel<br>11. Traditional Healer<br>12. No one else (Myself)<br>97. Other (specify) ______________	
	☐ HEALTH PROFESSIONAL Specify number: ____________<br>☐ OTHER (RELATION TO CAREGIVER) Specify number: ____________			Specify number: ____________
	☐ HEALTH PROFESSIONAL Specify number: ____________<br>☐ OTHER (RELATION TO CAREGIVER) Specify number: ____________			Specify number: ____________
	☐ HEALTH PROFESSIONAL Specify number: ____________<br>☐ OTHER (RELATION TO CAREGIVER) Specify number: ____________			Specify number: ____________
	☐ HEALTH PROFESSIONAL Specify number: ____________<br>☐ OTHER (RELATION TO CAREGIVER) Specify number: ____________			Specify number: ____________
	☐ HEALTH PROFESSIONAL Specify number: ____________<br>☐ OTHER (RELATION TO CAREGIVER) Specify number: ____________			Specify number: ____________
	☐ HEALTH PROFESSIONAL Specify number: ____________<br>☐ OTHER (RELATION TO CAREGIVER) Specify number: ____________			Specify number: ____________

30. Can you tell me the names of other western **anti**malaria medicines available in your community? **LIST BELOW ALL OTHER WESTERN antiMALARIA MEDICINES AVAILABLE IN THE COMMUNITY**

31.	Of the western **anti**malaria medicines you have mentioned, which do you think cures malaria the best in children child 5 years and under? ____
32.	Where did you learn this **anti**malaria medicine was the best?

1. Wall painting<br>2. Calendar<br>3. Drug shop provider<br>4. Health care staff	5. Medicine package<br>6. Leaflet (flyer)<br>7. Newspaper<br>8. Posters	9. Radio<br>10. Family members. Specify ________________<br>11. Neighbour	12. Other community members. Specify ________________<br>13. Fellow workers<br>14. Through training course	15. Through barazas<br>16. TV<br>17. No one else (Myself)<br>96. Other (specify) ________________

33.	Of the western **anti**malaria medicines you have mentioned, which one cures malaria the worst in children child 5 years and under? ____
34.	Where did you learn this **anti**malaria medicine was the worst?

1. Wall painting<br>2. Calendar<br>3. Drug shop provider<br>4. Health care staff	5. Medicine package<br>6. Leaflet (flyer)<br>7. Newspaper<br>8. Posters	9. Radio<br>10. Family members. Specify ________________<br>11. Neighbour	12. Other community members. Specify ________________<br>13. Fellow workers<br>14. Through training course	15. Through barazas<br>16. TV<br>17. No one else (Myself)<br>96. Other (specify) ________________

35.	If you had a choice, which western **anti**malaria medicine would you select first for this child's malaria? ____<br><br>Why? ____
36.	If you had a choice, which western **anti**malaria medicines would you select last for this child's malaria? ____<br><br>Why? ____
37.	Could you have independently decided to start this child on a different western **anti**malaria medicine or would you have required permission?
	1. Yes, can start independently 0. No, would require permission
38.	Who makes the final decision about which western **anti**malaria medicine your child can be started on?

**HEALTH PROFESSIONAL:**	**OTHER (RELATION TO CAREGIVER):**		
1. Doctor<br>2. Pharmacist<br>3. Nurse<br>96. Other (specify) ________________	4. Spouse<br>5. Father<br>6. Mother	7. Grandmother<br>8. In-Law<br>9. Sibling<br>10. Neighbour	11. Drug shop personnel<br>12. Traditional healer<br>13. No one else (Myself)<br>97. Other (specify) ________________________

39.	Which western **anti**malaria medicine does the government recommend for children child 5 years and under? **(CIRCLE FROM LIST BELOW or PICTURES)**

Amodiaquine	Artesunate	Clindamycin	Isoniazid	Pyrimethamine	Sulfalene
Arteether	Atovaquone	Dapsone	Lumefantrine	Pyronaridine	Sulfamethoxazole
Arteflene	Cloroquine	Dihydroartemisinin	Mefloquine	Quinidine	Sulfamethoxypyrazine
Artemether	Chlorproguanil	Doxycycline	Naphthoquine	Quinine	Sulfametopyrazine
Artemisinin	Cinchonidine	Halofantrine	Piperaquine	Rifampicin	Tafenoquine
Artemotil	Cinchonine	Hydroxychloroquine	Proguanil	Sulfadoxine	Tetracycline

Other: ____

40.	**IF CAREGIVER IS AWARE THE GOVERNMENT RECOMMENDS "ACT", ASK:** Where did you learn about what the government recommends?

1. Wall painting<br>2. Calendar<br>3. Drug shop provider<br>4. Health care staff	5. Medicine package<br>6. Leaflet (flyer)<br>7. Newspaper<br>8. Posters	9. Radio<br>10. Family members. Specify ________________<br>11. Neighbour	12. Other community members. Specify ________________<br>13. Fellow workers<br>14. Through training course	15. Through barazas<br>16. TV<br>17. No one else (Myself)<br>96. Other (specify) ________________

41.	In your opinion, how effective are the government recommended **anti**malaria medicines "ACT" compare to your "best" choice (**REFER TO Q.31**)?

1. Less effective<br>2. Same effectiveness	3. More effective<br>90. Don't know	95. Caregiver selected "ACT" as best **anti**malaria medicine

42.	**IF CAREGIVER *KNOWS* THAT THE GOVERNMENT RECOMMNENDS "ACT" (FROM Q.40), BUT THIS *WAS NOT* THE *VERY FIRST* antiMALARIA MEDICINE USED (REFER TO Q.27) IN THIS CHILD, ASK:**<br>Why did you not use "ACT" as the very first **anti**malaria medicine in this child?

1. Fever was not serious, waiting for fever to get worse before seeking treatment<br>2. Usually first wait and see if fever will go away	3. No money to buy "ACT"<br>4. No transportation to buy "ACT"<br>5. The place to obtain "ACT" was too far away	6. No one in the household had time to obtain "ACT"<br>7. Did not know where to go to get "ACT"	8. "ACT" not available at drug shop<br>9. "ACT" not available at health facility<br>10. Fever went away<br>11. No "ACT" at home	12. No drug shop open<br>13. No health facility open<br>14. Not recommended by ______________________<br>96. Other (specify): ______________________

43.	**IF CAREGIVER IS *NOT* AWARE THAT THE GOVERNMENT RECOMMENDS "ACT" AS FIRST LINE, THEN TELL CAREGIVER:**<br>The government now recommends "ACT" as first line.
44.	**REFER TO Q.35, DETERMINE IF "ACT" WAS SELECTED AS CAREGIVER'S FIRST CHOICE?**

1. Yes	0. No. **IF NO, ASK:** Knowing this, would your first **anti**malaria medicine choice be different?	
	1. Yes	0. No

45. Many different factors can prevent caregivers from getting the best western **anti**malaria medicines for a child. When you think your child is ill with malaria and want to get medicine for them, is each of the following a problem or no problem for you?

1. Knowing where to go	Problem		No problem
2. Getting permission to go	Problem		No problem
3. Having to find/take transportation	Problem		No problem
4. Access to health professional (ie: doctor, nurse)	Problem		No problem
5. Access to female health professional	Problem		No problem
6. Finding time	Problem		No problem
7. Availability of medicine	Problem		No problem
8. Health facility is too far	Problem		No problem
9. Getting money for medicine	Problem		No problem

10. Can you tell me two other factors that might be big problems for you

____

____

46. **INTERVIEWER SAY:** "I now want to ask you about each of the western **anti**malaria medicines that were given to this child".

**(DIRECTIONS TO INTERVIEWER): LIST OF ALL antiMALARIA MEDICATIONS FROM Q.27**	47. How long after the first symptom was this western **anti**malaria medicine started?	
	1. Immediately (within 6 hours)<br>2. Same day (within 1 day or 24 hours)<br>3. Next day (on the 2nd day or beyond 24 hours)	4. More than 2 days later (after 48 hours)<br>96. Other (specify)______________________
	1. Immediately (within 6 hours)<br>2. Same day (within 1 day or 24 hours)<br>3. Next day (on the 2nd day or beyond 24 hours)	4. More than 2 days later (after 48 hours)<br>96. Other (specify)______________________
	1. Immediately (within 6 hours)<br>2. Same day (within 1 day or 24 hours)<br>3. Next day (on the 2nd day or beyond 24 hours)	4. More than 2 days later (after 48 hours)<br>96. Other (specify)______________________
	1. Immediately (within 6 hours)<br>2. Same day (within 1 day or 24 hours)<br>3. Next day (on the 2nd day or beyond 24 hours)	4. More than 2 days later (after 48 hours)<br>96. Other (specify)______________________
	1. Immediately (within 6 hours)<br>2. Same day (within 1 day or 24 hours)<br>3. Next day (on the 2nd day or beyond 24 hours)	4. More than 2 days later (after 48 hours)<br>96. Other (specify)______________________
	1. Immediately (within 6 hours)<br>2. Same day (within 1 day or 24 hours)<br>3. Next day (on the 2nd day or beyond 24 hours)	4. More than 2 days later (after 48 hours)<br>96. Other (specify)______________________

48.	Can you tell me how soon after noticing fever in a child 5 years and under is it best to start a western **anti**malaria medicine?	
	1. Immediately (within 6 hours)<br>2. Same day (within 1 day or 24 hours)<br>3. Next day (on the 2nd day or beyond 24 hours)	4. More than 2 days later (after 48 hours)<br>96. Other (specify)_______________________________

49.	Where did you learn when it is best to start a western **anti**malaria medicine?				
	1. Wall painting<br>2. Calendar<br>3. Drug shop provider<br>4. Health care staff	5. Medicine package<br>6. Leaflet (flyer)<br>7. Newspaper<br>8. Posters	9. Radio<br>10. Family members. Specify<br>__________________<br>11. Neighbour	12. Other community members. Specify<br>__________________<br>13. Fellow workers<br>14. Through training course	15. Through barazas<br>16. TV<br>17. No one else (Myself)<br>96. Other (specify)<br>__________________

50.	In your opinion, how long is too long to start a western **anti**malaria medicine in a child 5 years and under?	
	1. Immediately (within 6 hours)<br>2. Same day (within 1 day or 24 hours)<br>3. Next day (2 days or beyond 24 hours)	4. More than 2 days later (after 48 hours)<br>96. Other (specify)_______________________________

51.	According to the government recommendations, when should a western **anti**malaria medicine be started after noticing the fever in children 5 years and under?		
	1. Immediately (within 6 hours)<br>2. Within 24 hours (1 day)<br>3. Within 48 hours (2 days)	4. After 2 days<br>5. Anytime<br>90. Don't Know	96. Other (specify)___________

52.	**IF THE CAREGIVER PROVIDES AN ANSWER FOR Q.51, ASK:** "Where have you seen or heard what the government recommends?"				
	1. Wall painting<br>2. Calendar<br>3. Drug shop provider<br>4. Health care staff	5. Medicine package<br>6. Leaflet (flyer)<br>7. Newspaper<br>8. Posters	9. Radio<br>10. Family members. Specify<br>__________________<br>11. Neighbour	12. Other community members. Specify<br>__________________<br>13. Fellow workers<br>14. Through training course	15. Through barazas<br>16. TV<br>17. No one else (Myself)<br>96. Other (specify)<br>__________________

53.	**IF FOR Q.51 THE CAREGIVER DOES NOT KNOW *or* GETS THE ANSWER WRONG, GIVE THE FOLLOWING INFORMATION:**<br><br>"The government recommends starting a western **anti**malaria medicine within 24 hours (or 1 day) of first noticing fever in children 5 years and under".

54.	In your opinion, is the government recommended start time of 24 hours for western **anti**malaria medicines realistic in children 5 years and under?	
	1. Yes	1. No.<br>Why? ____

55.	Can you independently decide <u>when</u> to start a western **anti**malaria medicine for your child or would you have required permission?	
	1. Yes	0. No

56.	Who makes the final decision <u>when</u> western **anti**malaria medicine can be started in your child?			
	**HEALTH PROFESSIONAL:**<br><br>1. Doctor<br>2. Pharmacist<br>3. Nurse<br>96. Other (specify) _________________	**OTHER (RELATION TO CAREGIVER):**<br><br>4. Spouse<br>5. Father<br>6. Mother	7. Grandmother<br>8. In-Law<br>9. Sibling<br>10. Neighbour	11. Drug shop personnel<br>12. Traditional healer<br>13. No one else (Myself)<br>97. Other (specify)<br>____________________________

57.	When do you think it is best to stop a western **anti**malaria medicine in children 5 years and under?			
	1. Within 1 day (24 hours)<br>1. Within 2 days (48 hours)<br>2. After 2 days later (after 48 hours)	2. If child has bad reaction<br>3. Took all the medicines that was recommended	4. Gave what I had<br>5. Symptoms got better<br>6. When there is no improvement	96. Other (specify) ___________

58.	Can you independently decide when to stop a western **anti**malaria medicine for your child?	
	1. Yes	0. No

<table>
<tr><td rowspan="3">LIST OF ALL antiMALARIA MEDICINES GIVEN TO THIS CHILD FROM Q.27</td><td colspan="7">59. INTERVIEWER SAY: “I now want to ask you <u>when you stopped</u> each of the western antimalaria medicine that was given to this child, starting with the first antimalaria medicine”</td></tr>
<tr><td>60. Can you tell me when this antimalaria medicine was stopped?
(SELECT ANSWER FROM LIST BELOW)</td><td>61. Why was this antimalaria medicine stopped?
(SELECT ANSWER FROM LIST BELOW)</td><td colspan="3">62. Who decided when to stop this antimalaria medicine?
(SELECT ANSWER FROM LIST BELOW)</td><td colspan="2">63. Did this child use all of this antimalaria medicine that was obtained?
(SELECT ANSWER FROM LIST BELOW)</td></tr>
<tr><td>2. Immediately (within 6 hours)
3. Within 1 day (24 hours)
4. Next day (within 2 days/48 hours)
5. More than 2 days (after 48 hours)
96. Other (Specify) ____________</td><td>1. Finished medication
2. Saved some for later
3. Was instructed
96. Other (Specify) ____________</td><td>HEALTH PROFESSIONAL:
1. Doctor
2. Pharmacist
3. Nurse
96. Other, (specify) ______________</td><td colspan="2">OTHER (RELATION TO CAREGIVE)
4. Spouse
5. Father
6. Mother
7. Grandmother
8. In-Law
9. Sibling
10. Neighbour
11. Drug shop personnel
12. Traditional Healer
13. No one else (Myself)
97. Other (specify) ____________</td><td>1. Yes
0. No.</td><td>IF NO, ASK: Why did the child not finish all the medicines?
1. Child had bad reaction
2. Took the amount that was recommended
3. Symptoms got better
4. When there is no improvement
96. Other (Specify) ___________</td></tr>
<tr><td>1.</td><td></td><td></td><td colspan="3"></td><td>Yes; No</td><td></td></tr>
<tr><td>2.</td><td></td><td></td><td colspan="3"></td><td>Yes; No</td><td></td></tr>
<tr><td>3.</td><td></td><td></td><td colspan="3"></td><td>Yes; No</td><td></td></tr>
<tr><td>4.</td><td></td><td></td><td colspan="3"></td><td>Yes; No</td><td></td></tr>
<tr><td>5.</td><td></td><td></td><td colspan="3"></td><td>Yes; No</td><td></td></tr>
<tr><td>6.</td><td></td><td></td><td colspan="3"></td><td>Yes; No</td><td></td></tr>
</table>

**LIST OF ALL MALARIA MEDICINES DETERMINED FROM Q27**	**Interviewer say:** "I now want to ask you about how you obtained each of the western **anti**malaria medicine that was given to this child"						
	64. Where was each of the medicines obtained from?<br>**(SELECT ANSWER FROM LIST BELOW)**		65. Who obtained the medicines? **(SELECT ANSWER FROM LIST BELOW)**	66. What was the one main reason for obtaining this medicine from this source?<br>**(SELECT ANSWER FROM LIST BELOW)**		67. **FOR EACH, IF A PUBLIC HEALTH FACILITY WAS NOT USED AS A SOURCE, ASK:** Why?<br>**(SELECT ANSWER FROM LIST BELOW)**	
	1. Community Health Worker<br>2. Drug shop<br>3. Public Health Facility<br>4. Neighbour<br>5. Pharmacy<br>6. Homestock, **IF HOMESTOCK, ASK** original source	7. Grocery store<br>8. Kiosk<br>9. Mobile providers<br>10. Private clinics/hospitals<br>11. Petrol stations<br>12. Traditional healer<br>96. Other (specify)	1. Myself<br>2. Spouse<br>3. Father<br>4. Mother<br>5. Grandmother<br>6. In-Law<br>7. Sibling<br>8. Neighbour<br>96. Other (specify) _______________	1. Close by/easy to reach<br>2. Reputation for quality treatment<br>3. No service cost<br>4. Availability for inexpensive medicine<br>5. Provides credit for purchases<br>6. Fast Service	7. Friendly Service<br>8. Felt illness was uncomplicated<br>9. Source equipped to handle severe illnesses<br>10. Qualified Health Professional<br>96. Other (specify): _______________	1. Far/not easy to reach<br>2. Reputation for poor quality treatment<br>3. Expensive service<br>4. Expensive medicine<br>5. No credit<br>6. Slow Service<br>7. Unfriendly Service<br>8. Felt illness was uncomplicated	9. No time to go<br>10. No transportation<br>11. Closed<br>12. Had medicines at home<br>13. No one available to take child<br>14. Advised to not go by _____________<br>96. Other (specify): _____________

68.	If you had a choice where would you go to obtain a western **anti**malaria medicine for this child? **(CIRCLE ALL THAT APPLY)**			
	1. Community Health Worker<br>2. Drug shop<br>3. Public Health Facility<br>4. Neighbour	5. Pharmacy<br>6. Homestock<br>7. Grocery store	8. Kiosk<br>9. Mobile providers<br>10. Private clinics/hospitals	11. Petrol stations<br>12. Traditional healer<br>96. Other (specify) ____________________

69.	Where can "ACT" be obtained in your community? **(CIRCLE ALL THAT APPLY)**			
	1. Community Health Worker<br>2. Drug shop<br>3. Public Health Facility<br>4. Neighbour	5. Pharmacy<br>6. Homestock<br>7. Grocery store<br>8. Kiosk	9. Mobile providers<br>10. Private clinics/hospitals<br>11. Petrol stations<br>12. Traditional healer	90. Don't know<br>96. Other (specify) ___________________

70.	Where have you seen or heard information where "ACT" can be obtained?				
	1. Wall painting<br>2. Calendar<br>3. Drug shop provider<br>4. Health care staff	5. Medicine package<br>6. Leaflet (flyer)<br>7. Newspaper<br>8. Posters	9. Radio<br>10. Family members. Specify ___________________<br>11. Neighbour	12. Other community members. Specify ___________________<br>13. Fellow workers<br>14. Through training course	15. Through barazas<br>16. TV<br>17. No one else (Myself)<br>96. Other (specify) ___________________

71.	Would you say "ACT" are easy to find in your community at any time when the need arises?	
	1. Yes	0. No

INTERVIEWER SAY: "I now want to ask you about <u>how</u> each of the western **anti**malaria medicine given to this child was <u>obtained</u>".

**LIST OF ALL MALARIA MEDICINES DETERMINED FROM Q27**	72. **REFER TO Q.65, IF CAREGIVER OBTAINED THE antiMALARIA, ASK**: Did you have money to pay for this medicine?					
	1. Yes	0. No				
	**IF YES, ASK:** Where would that money have come from?					
		1. Spouse<br>2. Father<br>3. Mother	4. Grandmother<br>5. In-Law<br>6. Sister	7. Brother<br>8. Neighbour	9. Drug shop personnel<br>10. Traditional Healer	11. No one else (Myself)<br>96. Other (specify) _______________
	Yes; No					
	Yes; No					
	Yes; No					
	Yes; No					
	Yes; No					

73.	Are "ACT" available in your community affordable for you? **(CIRCLE ALL THAT APPLY)**	
	1. Yes	0. No

74.	If you could obtain "ACT" from a shop for your child, how much could you afford to pay?<br><br>____

75.	Where are "ACT" available *<u>for free</u>* in your community? **(CIRCLE ALL THAT APPLY)**			
	1. Community Health Worker<br>2. Drug shop<br>3. Public Health Facility	4. Neighbour<br>5. Pharmacy<br>6. Homestock	7. Grocery store<br>8. Kiosk<br>9. Mobile providers<br>10. Private clinics/hospitals	11. Petrol stations<br>12. Traditional healer<br>90. Don't know<br>96. Other (specify) ____________________________

76.	Where can "ACT" be obtained *<u>on credit</u>* in your community? **(CIRCLE ALL THAT APPLY)**			
	1. Community Health Worker<br>2. Drug shop<br>3. Public Health Facility	4. Neighbour<br>5. Pharmacy<br>6. Homestock	7. Grocery store<br>8. Kiosk<br>9. Mobile providers<br>10. Private clinics/hospitals	11. Petrol stations<br>12. Traditional healer<br>90. Don't know<br>96. Other (specify) ____________________________

77.	In the last 6 months, have you ever kept western **anti**malaria medicines to use later just in case one of your children got malaria?	
	1. Yes	0. No

78. What type of western **anti**malaria medicines do you keep in your house? Can I see these? **(RECORD DRUG NAME – BRAND AND GENERIC, EXPIRY DATES)**			79. Did you purchase or borrow the western **anti**malaria medicines you keep in your house?		80. **ASK: WHERE antiMALARIA MEDICINES WERE PURCHASED or BORROWED FROM (SELECT FROM TABLE: LIST OF PROVIDERS BELOW).**
Brand Name	Generic Name	Expiry date (Month/Year)			
			Purchase	Borrow	
			Purchase	Borrow	
			Purchase	Borrow	
			Purchase	Borrow	
			Purchase	Borrow	
			Purchase	Borrow	
			Purchase	Borrow	
			Purchase	Borrow	
			Purchase	Borrow	
			Purchase	Borrow	
			Purchase	Borrow	
			Purchase	Borrow	

TABLE: LIST OF PROVIDERS

1. Community Health Worker<br>2. Drug shop<br>3. Public Health Facility	4. Neighbour<br>5. Pharmacy<br>6. Homestock	7. Grocery store<br>8. Kiosk<br>9. Mobile providers	10. Private clinics/hospitals<br>11. Petrol stations<br>12. Traditional healer

81.	What is the difference between good and poor quality western **anti**malaria medicines?<br>____				
82.	Where did you learn about poor quality western **anti**malaria medicines?				
	1. Wall painting<br>2. Calendar<br>3. Drug shop provider<br>4. Health care staff	5. Medicine package<br>6. Leaflet (flyer)<br>7. Newspaper<br>8. Posters	9. Radio<br>10. Family members. Specify __________________<br>11. Neighbour	12. Other community members. Specify __________________<br>13. Fellow workers<br>14. Through training course	15. Through barazas<br>16. TV<br>17. No one else (Myself)<br>96. Other (specify) __________________
83.	**IF FAKE AND EXPIRED ARE NOT MENTIONED, STATE THE FOLLOWING:**<br>**“**The government considers fake and expired western **anti**malaria medicines to be of poor quality”.				
84.	Have you ever been sold a poor quality western **anti**malaria medicine?				
	1. Yes	1. No			
85.	Do you know which outlets in your community have the best quality “ACT”? **(CIRCLE ALL THAT APPLY)**				
	1. Community Health Worker<br>2. Drug shop<br>3. Public Health Facility	4. Neighbour<br>5. Pharmacy<br>6. Homestock	7. Grocery store<br>8. Kiosk<br>9. Mobile providers<br>10. Private clinics/hospitals	11. Petrol stations<br>12. Traditional healer<br>90. Don’t know<br>96. Other (specify) ________________	
86.	Do you know which outlets in your community have the worst “ACT”? **(CIRCLE ALL THAT APPLY)**				
	1. Community Health Worker<br>2. Drug shop<br>3. Public Health Facility	4. Neighbour<br>5. Pharmacy<br>6. Homestock	7. Grocery store<br>8. Kiosk<br>9. Mobile providers<br>10. Private clinics/hospitals	11. Petrol stations<br>12. Traditional healer<br>90. Don’t know<br>96. Other (specify) ________________	

87. For each of the provider(s) you mentioned, I now want to ask you what information you received from each of these individuals. I will list different information, please state “yes or no” if you received this information
(DIRECTION TO INTERVIEWER): GO THROUGH EACH PROVIDER LISTED IN Q.80

	**Community Health Worker**	**Drug shop**	**Public Health Facility**	**Neighbour**	**Pharmacy**	**Homestock**	**Grocery Store**	**Kiosk**	**Mobile Provider**	**Private clinics / Hospitals**	**Petrol Stations**	**Traditional Healer**	**Other (specify) ____________________**
a. How malaria is caused	Y N	Y N	Y N	Y N	Y N	Y N	Y N	Y N	Y N	Y N	Y N	Y N	Y N
b. How malaria can be prevented	Y N	Y N	Y N	Y N	Y N	Y N	Y N	Y N	Y N	Y N	Y N	Y N	Y N
c. What is the best western **anti**malaria medicine for children	Y N	Y N	Y N	Y N	Y N	Y N	Y N	Y N	Y N	Y N	Y N	Y N	Y N
d. How to administer the medicine	Y N	Y N	Y N	Y N	Y N	Y N	Y N	Y N	Y N	Y N	Y N	Y N	Y N
e. The importance of completing the full course of medicine	Y N	Y N	Y N	Y N	Y N	Y N	Y N	Y N	Y N	Y N	Y N	Y N	Y N
f. What to do if the child vomits the medicine	Y N	Y N	Y N	Y N	Y N	Y N	Y N	Y N	Y N	Y N	Y N	Y N	Y N
g. What to do if the child does not get better	Y N	Y N	Y N	Y N	Y N	Y N	Y N	Y N	Y N	Y N	Y N	Y N	Y N
h. What foods to give the child with the medicine	Y N	Y N	Y N	Y N	Y N	Y N	Y N	Y N	Y N	Y N	Y N	Y N	Y N
i. Storing the medicine at home	Y N	Y N	Y N	Y N	Y N	Y N	Y N	Y N	Y N	Y N	Y N	Y N	Y N
j. Did they offer you any other advice?	Y N	Y N	Y N	Y N	Y N	Y N	Y N	Y N	Y N	Y N	Y N	Y N	Y N
k. What additional information would you have liked to have received?													
l. Compared to doctor/ nurse, how knowledgeable was this provider?	Y N	Y N	Y N	Y N	Y N	Y N	Y N	Y N	Y N	Y N	Y N	Y N	Y N
m. Where do you think this provider learned how to treat malaria?	Y N	Y N	Y N	Y N	Y N	Y N	Y N	Y N	Y N	Y N	Y N	Y N	Y N

88. When you obtain a western **anti**malaria medicine for your child, what kind of additional information would you like to receive from the person who is giving you the medicine?

____ ____ ____ ____

____ ____ ____ ____

89.	If you had a choice to go anywhere to obtain advice on how to treat malaria in your children 5 years of age and under, who would you go to <u>first</u>?

	**HEALTH PROFESSIONAL:**	**OTHER (RELATION TO CAREGIVER):**			
	1. Doctor<br>2. Pharmacist<br>3. Nurse<br>96. Other (specify) ________________	4. Spouse<br>5. Father<br>6. Mother	7. Grandmother<br>8. In-Law<br>9. Sibling	10. Neighbour<br>11. Drug shop personnel<br>12. Traditional healer	13. No one else (Myself)<br>97. Other (specify) _______________

90.	**IF SPOUSE NOT MENTIONED ASK:**<br>You have not mentioned your spouse, why? ____
91.	Many different factors can prevent caregivers from getting <u>advice</u> for treating malaria in children 5 years and under. When you think your child is ill with malaria and want to get the best advice, is each of the following a problem or no problem for you?

	a. Knowing where to go	Problem		No problem
	b. Getting permission to go	Problem		No problem
	c. Having to take transportation	Problem		No problem
	d. Access to health professional (ie: doctor, nurse)	Problem		No problem
	e. Access to female health professional	Problem		No problem
	f. Finding time	Problem		No problem
	g. Resources within the community	Problem		No problem
	h. Can you tell me <u>two</u> other factors that might be a <u>big</u> problem for you:<br>____<br>____			

PART 3. FOR CHILDREN WHO *DID NOT RECEIVE ANY* ANTIMALARIA MEDICINES (May Have Received Other Western Medicines) From Q. 27

92. Even though your child did not receive any antimalarial medicines, can you tell me the names of all western **anti**malaria medicines available in your community? **(DIRECTION TO INTERVIEWER: USE LIST OF antiMALARIA MEDICINES FROM TABEL BELOW or PICTURES**

TABLE: WESTERN antiMALARIA MEDICINE LIST

Amodiaquine	Artesunate	Clindamycin	Isoniazid	Pyrimethamine	Sulfalene
Arteether	Atovaquone	Dapsone	Lumefantrine	Pyronaridine	Sulfamethoxazole
Arteflene	Cloroquine	Dihydroartemisinin	Mefloquine	Quinidine	Sulfamethoxypyrazine
Artemether	Chlorproguanil	Doxycycline	Naphthoquine	Quinine	Sulfametopyrazine
Artemisinin	Cinchonidine	Halofantrine	Piperaquine	Rifampicin	Tafenoquine
Artemotil	Cinchonine	Hydroxychloroquine	Proguanil	Sulfadoxine	Tetracycline

Other: ____

93. Of the western **anti**malaria medicines you have mentioned, which do you think cures malaria the <u>best</u> in children 5 years of age and under?
____

94. Where did you learn this **anti**malaria medicine was the <u>best</u>?

1. Wall painting<br>2. Calendar<br>3. Drug shop provider<br>4. Health care staff	5. Medicine package<br>6. Leaflet (flyer)<br>7. Newspaper<br>8. Posters	9. Radio<br>10. Family members. Specify ___________________<br>11. Neighbour	12. Other community members. Specify ___________________<br>13. Fellow workers<br>14. Through training course	15. Through barazas<br>16. TV<br>17. No one else (Myself)<br>96. Other (specify) ___________________

95. Of the western **anti**malaria medicines you have mentioned, which one cures malaria the <u>worst</u> in children 5 years and under?
____

96. Where did you learn this **anti**malaria medicine was the worst?

1. Wall painting<br>2. Calendar<br>3. Drug shop provider<br>4. Health care staff	5. Medicine package<br>6. Leaflet (flyer)<br>7. Newspaper<br>8. Posters	9. Radio<br>10. Family members. Specify ___________________<br>11. Neighbour	12. Other community members. Specify ___________________<br>13. Fellow workers<br>14. Through training course	15. Through barazas<br>16. TV<br>17. No one else (Myself)<br>96. Other (specify) ___________________

97. If you had a choice, which of these western **anti**malaria medicines would you select <u>first</u> for this child's malaria?
____ Why? ____

98. If you had a choice, which of these western **anti**malaria medicines would you select <u>last</u> for this child's malaria?
____ Why? ____

99. Can you independently decide to start this child on a western **anti**malaria medicine or would you require permission?

1. Yes	0. No

100.	Who makes the decision about which western **anti**malaria medicine your child can be started on?

	HEALTH PROFESSIONAL:	OTHER (RELATION TO CAREGIVER):		
	1. Doctor<br>2. Pharmacist<br>3. Nurse<br>96. Other (specify) ________________	4. Spouse<br>5. Father<br>6. Mother	7. Grandmother<br>8. In-Law<br>9. Sibling<br>10. Neighbour	11. Drug shop personnel<br>12. Traditional healer<br>13. No one else (Myself)<br>97. Other (specify)<br>__________________________

101.	What western **anti**malaria medicines does the government recommend for children 5 years and under? **(CIRCLE ALL THAT APPLY)**

Amodiaquine	Artesunate	Clindamycin	Isoniazid	Pyrimethamine	Sulfalene
Arteether	Atovaquone	Dapsone	Lumefantrine	Pyronaridine	Sulfamethoxazole
Arteflene	Cloroquine	Dihydroartemisinin	Mefloquine	Quinidine	Sulfamethoxypyrazine
Artemether	Chlorproguanil	Doxycycline	Naphthoquine	Quinine	Sulfametopyrazine
Artemisinin	Cinchonidine	Halofantrine	Piperaquine	Rifampicin	Tafenoquine
Artemotil	Cinchonine	Hydroxychloroquine	Proguanil	Sulfadoxine	Tetracycline

Other: ____

102.	**IF CAREGIVER IS AWARE THAT THE GOVERNMENT RECOMMENDS "ACT", Ask:** Where did you learn about what the government recommends?

1. Wall painting<br>2. Calendar<br>3. Drug shop provider<br>4. Health care staff	5. Medicine package<br>6. Leaflet (flyer)<br>7. Newspaper<br>8. Posters	9. Radio<br>10. Family members. Specify<br>_________________<br>11. Neighbour	12. Other community members. Specify<br>_________________<br>13. Fellow workers<br>14. Through training course	15. Through barazas<br>16. TV<br>17. No one else (Myself)<br>96. Other (specify)<br>_________________

103.	**IF CAREGIVER IS *NOT* AWARE THAT THE GOVERNMENT RECOMMENDS "ACT" AS FIRST LINE, THEN TELL CAREGIVER:**<br>"The government now recommends "ACT" as first line"
104.	In your opinion, how effective are "ACT", compare to your best choice **(REFER TO Q.93)**?

1. Less effective<br>2. Same effectiveness	3. More effective<br>90. Don't know	95. Caregiver selected "ACT" as best antimalaria

105.	**REFER TO Q.97, WAS "ACT" SELECTED AS CAREGIVER'S FIRST CHOICE WESTERN antiMALARIA MEDICNE?**

1. Yes,	0. No,<br>**IF NO, ASK:** Knowing this, would your first **anti**malaria choice be different?	
	1. Yes	0. No

106.	Can you tell me how soon after noticing fever in a child 5 years and under is it best to start a western **anti**malaria medicine?

1. Immediately (within 6 hours)<br>2. Same day (within 1 day or 24 hours)<br>3. Next day (on the 2nd day or beyond 24 hours)	4. More than 2 days later (after 48 hours)<br>96. Other (specify)_____________________________

107.	Where did you learn when it is best to start a western **anti**malaria medicine?

1. Wall painting<br>2. Calendar<br>3. Drug shop provider<br>4. Health care staff	5. Medicine package<br>6. Leaflet (flyer)<br>7. Newspaper<br>8. Posters	9. Radio<br>10. Family members. Specify<br>_________________<br>11. Neighbour	12. Other community members. Specify<br>_________________<br>13. Fellow workers<br>14. Through training course	15. Through barazas<br>16. TV<br>17. No one else (Myself)<br>96. Other (specify)<br>_________________

108.	In your opinion, how long is too long to start a western **anti**malaria medicine in a child 5 years and under?

1. Immediately (within 6 hours)<br>2. Same day (within 1 day or 24 hours)<br>3. Next day (2 days or beyond 24 hours)	4. More than 2 days later (after 48 hours)<br>96. Other (specify)_____________________________

109.	According to the government recommendations, when should a western **anti**malaria medicine be started after noticing a fever in children 5 years and under?

1. Immediately (within 6 hours)<br>2. Same day (within 1 day or 24 hours)<br>3. Next day (on the 2nd day or beyond 24 hours)	4. More than 2 days later (after 48 hours)<br>96. Other (specify)_____________________________

110.	**IF THE CAREGIVER VOLUNTEERS AN ANSWER FOR Q.109, ASK:** "Where have you seen or heard what the government recommends"?

1. Wall painting<br>2. Calendar<br>3. Drug shop provider<br>4. Health care staff	5. Medicine package<br>6. Leaflet (flyer)<br>7. Newspaper<br>8. Posters	9. Radio<br>10. Family members. Specify ________________<br>11. Neighbour	12. Community members. Specify ________________<br>13. Fellow workers<br>14. Through training course	15. Through barazas<br>16. TV<br>17. No one else (Myself)<br>96. Other (specify) __________________

111.	**IF THE CAREGIVER DOES NOT KNOW WHAT THE GOVERNMENT RECOMMENDS or GETS THE ANSWER IN Q.109 WRONG, GIVE THE FOLLOWING INFORMATION:**<br>"The government recommends starting an **anti**malaria medicine within 24 hours (or 1 day) of first noticing fever in children 5 years and under."
112.	In your opinion, is the government recommended start time of 24 hours for western **anti**malaria medicines realistic in children 5 years and under?

1. Yes	0. No, Why? ____

113.	Can you independently decide <u>when to start</u> a western **anti**malaria medicine for this child or would you require permission?

1. Yes	0. No

114.	Who makes the final decision <u>when</u> western **anti**malaria medicine can be <u>started</u> in your child?

**HEALTH PROFESSIONAL:**	**OTHER (RELATION TO CAREGIVER):**		
1. Doctor<br>2. Pharmacist<br>3. Nurse<br>96. Other (specify) _________________	4. Spouse<br>5. Father<br>6. Mother	7. Grandmother<br>8. In-Law<br>9. Sibling<br>10. Neighbour	11. Drug shop personnel<br>12. Traditional healer<br>13. No one else (Myself)<br>97. Other (specify) ___________________________

115.	When do you think it is best to stop a western **anti**malaria medicine in children 5 years and under?

1. Within 1 day (24 hours)<br>2. Within 2 days (48 hours)<br>3. After 2 days later (after 48 hours)	4. If child has bad reaction<br>5. Took the amount that was recommended	6. Gave what I had<br>7. Symptoms got better	8. When there is no improvement<br>96. Other (specify)___________

116.	If a western **anti**malaria medicine is started in this child, who makes the final decision about when to stop the medicine?

**HEALTH PROFESSIONAL:**	**OTHER (RELATION TO CAREGIVER):**		
1. Doctor<br>2. Pharmacist<br>3. Nurse<br>96. Other (specify) _________________	4. Spouse<br>5. Father<br>6. Mother	7. Grandmother<br>8. In-Law<br>9. Sibling<br>10. Neighbour	11. Drug shop personnel<br>12. Traditional healer<br>13. No one else (Myself)<br>97. Other (specify) ___________________________

117.	Can you independently decide when to stop a western **anti**malaria medicine for this child if one was started?

1. Yes	0. No

118.	Where do you normally obtain western **anti**malaria medicines for this child?

1. Community Health Worker<br>2. Drug shop<br>3. Public Health Facility	4. Neighbour<br>5. Pharmacy<br>6. Homestock	7. Grocery store<br>8. Kiosk<br>9. Mobile providers<br>10. Private clinics/hospitals	11. Petrol stations<br>12. Traditional healer<br>90. Don't know<br>96. Other (specify) ____________________

119.	What is the <u>one main</u> reason for obtaining western **anti**malaria medicines from this source?

1. Close by/easy to reach<br>2. Reputation for quality treatment<br>3. No service cost	4. Availability for inexpensive medicine<br>5. Provides credit for purchases<br>6. Fast Service	7. Friendly Service<br>8. Felt illness was uncomplicated	9. Source equipped to handle severe illness<br>10. Qualified Health Professional<br>96. Other (specify): _____________________________

120.	**IF A PUBLIC HEALTH FACILITY IS NOT STATED IN Q.118 FOR OBTAINING A WESTERN ANTIMALARIA MEDICINE, THEN ASK** why? **(CIRCLE ALL THAT APPLY)**

1. Far/not easy to reach<br>2. Reputation for poor quality treatment<br>3. Expensive service	4. Expensive medicine<br>5. No credit<br>6. Slow service<br>7. Unfriendly Service	8. Felt illness was uncomplicated<br>9. No time to go<br>10. No transportation Closed<br>11. Had medicines at home	12. No one available to take child<br>13. Advised to not go by ___________________________________<br>96. Other (specify): ______________________

121.	Who normally obtains western **anti**malaria medicines for this child? **(CIRCLE ALL THAT APPLY)**			
	1. Myself<br>2. Spouse<br>3. Father	4. Mother<br>5. Grandmother	6. In-Law<br>7. Sibling	8. Neighbour<br>96. Other (specify) ________________
122.	If you had a choice where would you go to obtain a western **anti**malaria medicine for this child? **(CIRCLE ALL THAT APPLY)**			
	1. Community Health Worker<br>2. Drug shop<br>3. Public Health Facility	4. Neighbour<br>5. Pharmacy<br>6. Homestock	7. Grocery store<br>8. Kiosk<br>9. Mobile providers<br>10. Private clinics/hospitals	11. Petrol stations<br>12. Traditional healer<br>96. Other (specify)<br>________________________________
123.	Where can "ACT" be obtained in your community? **(CIRCLE ALL THAT APPLY)**			
	1. Community Health Worker<br>2. Drug shop<br>3. Public Health Facility	4. Neighbour<br>5. Pharmacy<br>6. Homestock	7. Grocery store<br>8. Kiosk<br>9. Mobile providers<br>10. Private clinics/hospitals	11. Petrol stations<br>12. Traditional healer<br>96. Other (specify)<br>________________________________

124.	Where have you seen or heard information where "ACT" can be obtained?				
	1. Wall painting<br>2. Calendar<br>3. Drug shop provider<br>4. Health care staff	5. Medicine package<br>6. Leaflet (flyer)<br>7. Newspaper<br>8. Posters	9. Radio<br>10. Family members. Specify ____________________<br>11. Neighbour	12. Other community members. Specify ____________________<br>13. Fellow workers<br>14. Through training course	15. Through barazas<br>16. TV<br>17. No one else (Myself)<br>96. Other (specify) ____________________

125.	Would you say "ACT" are easy to find in your community at any time when the need arises?	
	1. Yes	0. No
126.	In the last 6 months, have you ever kept western **anti**malaria medicines to use later just in case one of your children got malaria?	
	1. Yes	0. No

127. What type of western **anti**malaria medicines do you keep in your house? Can I see these? **(RECORD DRUG NAME – BRAND AND GENERIC, EXPIRY DATES)**			128. Did you purchase or borrow the western **anti**malaria medicines you keep in your house?		129. **ASK WHERE FROM, SELECT FROM TABLE: LIST OF PROVIDERS**
Brand Name	Generic Name	Expiry date (Month /Year)			
			Purchase	Borrow	
			Purchase	Borrow	
			Purchase	Borrow	
			Purchase	Borrow	
			Purchase	Borrow	
			Purchase	Borrow	
			Purchase	Borrow	
			Purchase	Borrow	
			Purchase	Borrow	
			Purchase	Borrow	

TABLE: LIST OF PROVIDERS

1. Community Health Worker<br>2. Drug shop<br>3. Public Health Facility	4. Neighbour<br>5. Pharmacy<br>6. Homestock	7. Grocery store<br>8. Kiosk<br>9. Mobile providers	10. Private clinics/hospitals<br>11. Petrol stations<br>12. Traditional healer

130.	If you were to obtain a western **anti**malaria medicine where would the money have to come from? **(CIRCLE ALL THAT APPLY)**			
	1. Myself<br>2. Spouse<br>3. Father	4. Mother<br>5. Grandmother<br>6. In-Law	7. Sibling<br>8. Neighbour<br>9. Drug shop personnel	10. Traditional healer<br>96. Other (specify)<br>________________________________
131.	Are "ACT" available in your community affordable for you?			
	1. Yes	0. No		
132.	If you could obtain "ACT" from a shop for this child, how much could you afford to pay? ____			

133.	Where are "ACT" available *for free* in your community? **(CIRCLE ALL THAT APPLY)**
	1. Community Health Worker<br>2. Drug shop<br>3. Public Health Facility<br>4. Neighbour<br>5. Pharmacy<br>6. Homestock<br>7. Grocery store<br>8. Kiosk<br>9. Mobile providers<br>10. Private clinics/hospitals<br>11. Petrol stations<br>12. Traditional healer<br>90. Don't Know<br>96. Other (specify) ___________________________________
134.	Where can "ACT" be obtained *on credit* in your community? **(CIRCLE ALL THAT APPLY)**
	1. Community Health Worker<br>2. Drug shop<br>3. Public Health Facility<br>4. Neighbour<br>5. Pharmacy<br>6. Homestock<br>7. Grocery store<br>8. Kiosk<br>9. Mobile providers<br>10. Private clinics/hospitals<br>11. Petrol stations<br>12. Traditional healer<br>90. Don't know<br>96. Other (specify) ________________________________
135.	What is the difference between good and poor quality western **anti**malaria medicines?<br>____
136.	Where did you learn about poor quality western **anti**malaria medicines?
	1. Wall painting<br>2. Calendar<br>3. Drug shop provider<br>4. Health care staff<br>5. Medicine package<br>6. Leaflet (flyer)<br>7. Newspaper<br>8. Posters<br>9. Radio<br>10. Family members. Specify __________________<br>11. Neighbour<br>12. Other community members. Specify __________________<br>13. Fellow workers<br>14. Through training course<br>15. Through barazas<br>16. TV<br>17. No one else (Myself)<br>96. Other (specify) _________________
137.	**IF FAKE AND EXPIRED ARE NOT MENTIONED, STATE THE FOLLOWING:**<br>**"**The government considers fake and expired western **anti**malaria medicines to be of poor quality."
138.	Have you ever been sold a poor quality western **anti**malaria medicine?
	1. Yes<br>0. No
139.	Do you know which outlets in your community have the best quality "ACT"? **(CIRCLE ALL THAT APPLY)**
	1. Community Health Worker<br>2. Drug shop<br>3. Public Health Facility<br>4. Neighbour<br>5. Pharmacy<br>6. Homestock<br>7. Grocery store<br>8. Kiosk<br>9. Mobile providers<br>10. Private clinics/hospitals<br>11. Petrol stations<br>12. Traditional healer<br>90. Don't Know<br>96. Other (specify) ________________________________
140.	Do you know which outlets in your community have the worst "ACT"? **(CIRCLE ALL THAT APPLY)**
	1. Community Health Worker<br>2. Drug shop<br>3. Public Health Facility<br>4. Neighbour<br>5. Pharmacy<br>6. Homestock<br>7. Grocery store<br>8. Kiosk<br>9. Mobile providers<br>10. Private clinics/hospitals<br>11. Petrol stations<br>12. Traditional healer<br>90. Don't Know<br>96. Other (specify) _____________________
141.	Many different factors can prevent caregivers from getting the best western **anti**malaria medicines for a child. When you think your child is ill with malaria and want to get medicine for them, is each of the following a problem or no problem for you?

	a. Knowing where to go	Problem		No problem
	b. Getting permission to go	Problem		No problem
	c. Having to take transportation	Problem		No problem
	d. Access to health professional (ie: doctor, nurse)	Problem		No problem
	e. Access to female health professional	Problem		No problem
	f. Finding time	Problem		No problem
	g. Availability of medicine	Problem		No problem
	h. Health facility is too far	Problem		No problem
	i. Getting money for medicine	Problem		No problem
	j. Can you tell two other factors that might be a big problem<br>____<br>____			

142.	When you obtain a western **anti**malaria medicine for your child, what kind of information would you like to receive from the person who is giving you the medicine?								
How malaria is caused	How malaria can be prevented	What is the best western malaria medicine for children under 5 years of age?	How to administer the western malaria medicine to your child at home	The importance of completing the full course of western malaria medicine	What to do if the child vomits the western malaria medicine after the medicine has been swallowed?	What to do if the child does not get better following the treatment	What foods to give the child with the western malaria medicine	How to store the western malaria medicine at home	Other, Specify ___________

143.	Compared to a doctor or nurse, how knowledgeable about treating malaria is the person where you normally go to obtain your western **anti**malaria medicines?		
	1. Less knowledge	2. Same knowledge	3. More knowledge
144.	Where do you think this person who gives you the western **anti**malaria medicines has learned about how to treat malaria? ____		
145.	If you had a choice to go anywhere to obtain advice on how to treat malaria in this child, who would you go to <u>first</u>?		

	**NON-HEALTH PROFESSIONAL:**<br>1. Nobody (Myself)<br>2. Spouse<br>3. Father	4. Mother<br>5. Grandmother<br>6. In-Law<br>7. Sibling	8. Neighbour<br>9. Drug shop personnel<br>10. Traditional Healer<br>96. Other (specify) _______________________	**HEALTH PROFESSIONAL:**<br>11. Doctor<br>12. Pharmacist<br>13. Nurse<br>96. Other (specify) _________________
146.	**IF SPOUSE NOT MENTIONED ASK:** You have not mentioned your spouse, why?			

147.	Many different factors can prevent caregivers from getting <u>advice</u> for treating malaria in children 5 years and under. When you think your child is ill with malaria and want to get the best advice, is each of the following a problem or no problem for you?			
	a. Knowing where to go	Problem		No problem
	b. Getting permission to go	Problem		No problem
	c. Having to take transportation	Problem		No problem
	d. Access to health professional (ie: doctor, nurse)	Problem		No problem
	e. Access to female health professional	Problem		No problem
	f. Finding time	Problem		No problem
	g. Resources within the community	Problem		No problem
	h. Can you tell <u>two</u> other factors that might be a <u>big</u> problem for you:<br>____<br>____			

PART 4. PREVENTION QUESTIONS FOR ALL CAREGIVERS

148.	How many mosquito nets does your household have? ____			
149.	Who slept under the mosquito nets last night? _______________________________________			
150.	Which time of the year do you use the mosquito nets?			
	1. During the dry season	2. During the rainy season	3. Throughout the year	4. Not used
151.	**IF THERE ARE MOSQUITO NETS, ASK TO SEE THE NETS and LIST BELOW: OBSERVE OR ASK THE BRAND(s)** **<u>LIST:</u> 1. Permanent" net, 2. Brand A, 3. Brand B, 4. Brand C, 5. Brand D, 6. DK brand, 7. Pretreated net; 8. Other (specify) _____**	152. **FOR EACH NET, OBSERVE IF ALL NETS ARE HANGING**	153. **FOR EACH NET ASK:** How many months ago was the net last soaked or dipped?	
		Yes No	_____ Months; ______Years; Not sure	
		Yes No	_____ Months; ______Years; Not sure	
		Yes No	_____ Months; ______Years; Not sure	
		Yes No	_____ Months; ______Years; Not sure	
		Yes No	_____ Months; ______Years; Not sure	
		Yes No	_____ Months; ______Years; Not sure	
		Yes No	_____ Months; ______Years; Not sure	
		Yes No	_____ Months; ______Years; Not sure	
154.	Do any of your nets have holes in them?			
	1. Yes	0. No		
155.	How many months ago were the interior walls of your house sprayed? _____ Months; ______Years; Not sure			
156.	Who sprayed the walls in your house?			
	1. Government worker/program<br>2. Household member<br>3. Private Company	90. Don't know<br>96. Other (specify) _______________		

IF YOU SUSPECT FEVER or MALARIA IN YOUR CHILD, HOW CERTAIN ARE YOU THAT:	No Certain at all	So-so Certain	Completely Certain
157. You can recognize fever in your child when your child has fever?			
158. You can recognize malaria in your child when your child has malaria?			
159. You would know where to obtain the best advice for treating your child?			
160. You would be able to obtain the government recommended "ACT" to treat your child's malaria?			
161. You would know when to start the western **anti**malaria medicine for your child?			
162. You would know how often each day to give the western **anti**malaria medicine to your child?			
163. You would know how to give the western **anti**malaria medicine to your child?			
164. You would know if your child has recovered from malaria?			
165. You would know when to stop giving the western **anti**malaria medicine to your child?			

**(DIRECTIONS FOR INTERVIEWER): SAY TO THE CAREGIVER:**<br>"I now will ask you questions about Beliefs related to Malaria from 3 different viewpoints, starting with What do most people in this community believe, then What does your family believe, and ending with What do you personally believe. For each statement, tell me who thinks it is true and who thinks it is false."	**What do most people in this community believe?**		**What does your family believe?**		**What do you personally believe?**	
166. Malaria is totally preventable.	True	False	True	False	True	False
167. It is possible to cure all children from malaria.	True	False	True	False	True	False
168. No matter what you do, some children will die from malaria.	True	False	True	False	True	False
169. Children almost never die from malaria.	True	False	True	False	True	False
170. Left untreated, most children will die from malaria.	True	False	True	False	True	False
171. Some children can be cured from malaria using only home remedies.	True	False	True	False	True	False
172. Some children can be cured from malaria using only traditional medicines.	True	False	True	False	True	False
173. It is okay to first try to treat malaria at home before going to see a doctor or a nurse.	True	False	True	False	True	False
174. It is okay to use western medicine only if traditional medicines do not work.	True	False	True	False	True	False
175. It is okay to use a traditional medicine before trying western medicines.	True	False	True	False	True	False
176. Sometimes it is okay not to use western medicines to treat malaria.	True	False	True	False	True	False
177. Traditional medicines are best for curing malaria.	True	False	True	False	True	False
178. Western medicines are always best for curing malaria.	True	False	True	False	True	False
179. It is best to start a western **anti**malaria medicine within one day after noticing fever in your child.	True	False	True	False	True	False
180. A blood test is necessary to confirm malaria before starting any treatment.	True	False	True	False	True	False
181. It is okay to stop a western **anti**malaria medicine as soon as the fever goes away.	True	False	True	False	True	False
182. It is important for your child to take all the western **anti**malaria medicines s/he has been given.	True	False	True	False	True	False

183. Government clinics are the best place to obtain western **anti**malaria medicines.	True	False	True	False	True	False
184. Most malaria is caused by mosquito bites.	True	False	True	False	True	False
185. Burning mosquito oils is best for preventing malaria.	True	False	True	False	True	False

186. What is the main reason that children die from malaria?

What do most people in this community believe?	What does your family believe?	What do you believe?
______________________________	______________________________	______________________________
______________________________	______________________________	______________________________
______________________________	______________________________	______________________________

SAY TO CAREGIVER: "For the next statements tell me which medicines are the best or worst, and who thinks so"

187. Which western **anti**malaria medicines are best and worst for curing malaria?	**What do most people in this community believe?**		**What does your family believe?**		**What do you personally believe?**	
a. Loose Tablets?	Best	Worst	Best	Worst	Best	Worst
b. Syrups?	Best	Worst	Best	Worst	Best	Worst
c. Capsules or Tablets from bottles?	Best	Worst	Best	Worst	Best	Worst
d. Medicines in packages?	Best	Worst	Best	Worst	Best	Worst
e. Powder to mix with water?	Best	Worst	Best	Worst	Best	Worst

188.	What is your top <u>two</u> preferred sources of information? How accessible is each? **(SELECT FROM TABLE: LIST OF INFORMATION SOURCES)**			
	a. PREFERENCE #1	Very Accessible	So-So Accessible	Not Accessible
	b. PREFERENCE #1	Very Accessible	So-So Accessible	Not Accessible

LIST OF INFORMATION SOURCES

1. Wall painting<br>2. Calendar<br>3. Drug shop provider<br>4. Health care staff	5. Medicine package<br>6. Leaflet (flyer)<br>7. Newspaper<br>8. Posters	9. Radio<br>10. Family members. Specify ________________<br>11. Neighbour	12. Other community members. Specify ________________<br>13. Fellow workers<br>14. Through training course	15. Through barazas<br>16. TV<br>17. No one else (Myself)<br>97. Other (specify) ________________

189.	What is the distance from home to the nearest public health facility?			
	1. <1/2km<br>2. ½ to < 1km	3. 1km – 5km<br>4. More than 5km	5. Can you tell me the exact distance? ____________ km	
190.	What is the distance from home to the nearest drug shop (regulated)?			
	1. <1/2km<br>2. ½ to < 1km	3. 1km – 5km<br>4. More than 5km	5. Can you tell me the exact distance? ____________ km	
191.	What is the distance from home to the nearest drug shop (unregulated)?			
	1. <1/2km<br>2. ½ to < 1km	3. 1km – 5km<br>4. More than 5km	5. Can you tell me the exact distance? ____________ km	
192.	What is the distance from home to the nearest traditional healer?			
	1. <1/2km<br>2. ½ to < 1km	3. 1km – 5km<br>4. More than 5km	5. Can you tell me the exact distance? ____________ km	
193.	In your opinion, how easy to reach are public health facilities (clinics or hospitals) in your community?			
	1. Very Easy	2. So-so	3. Very Difficult	90. Don't Know
	**DIRECTIONS FOR INTERVIEWERS: FOR THE NEXT 12 QUESTIONS, OBSERVE AS MUCH AS YOU CAN DO NOT ASK UNLESS YOU MUST**			
194.	What type of dwelling does the household live in?			
	1. Hut<br>2. Independent house	3. Muzigo<br>4. Shared house	5. Uniport<br>6. Other (specify) _____________________	
195.	How many people usually sleep in this household?<br>____			
196.	How many rooms in this household are used for sleeping?<br>____			
197.	What type of roof does the house have?			
	1. Cement<br>2. Grass or thatch	3. Iron sheets or tin<br>4. Mud	5. Shingles<br>6. Tiles	7. No Roof<br>8. Other (specify)
198.	What is the main material of the floor?			
	1. Finished floor: cement, tiles, linoleum, carpet, polished wood, or stones	2. Natural floor: earth, sand, dung, or clay	3. Rudimentary floor: wooden planks, palm, or bamboo<br>4. Floating house	5. Iron sheets<br>96. Other (specify) _________________
199.	What is the main source of water for drinking in this household?			
	1. Borehole or well<br>2. Bottled water	3. Brought-in (jerry can, tanker truck)<br>4. Piped or tap water	5. Rain water<br>6. Surface water (stream, river, pond, lake, dam, spring)	96. Other (specify) _______________________
200.	What kind of toilet facilities does this household use?			
	1. Bucket<br>2. Bush/ field/ forest	3. Composting toilet<br>4. Flush toilet	5. Traditional pit toilet/ latrine<br>6. Ventilated improved pit latrine	96. Other (specify)
201.	Is this toilet shared with other households?			
	1. Yes		0. No	
202.	What types of walls are there in the house?			
	1. Cardboard<br>2. Clay or mud	3. Iron sheets<br>4. Palm/ bamboo/ thatch/ straw	5. Wood<br>6. No walls	96. Other (specify)

203.	What is the main type of fuel used by this household for cooking?			
	1. Agricultural crop<br>2. Charcoal<br>3. Coal<br>4. Dung	5. Electricity<br>6. Firewood<br>7. Kerosene/ paraffin	8. Natural gas/ liquid petroleum gas (LPG)<br>9. Solar<br>10. Straws/ shrubs/ grass	11. No food cooked in household<br>96. Other (specify) ______________________

204.	How many of the following livestock does this household have?		
	1. Chicken<br>2. Cow	3. Duck	4. Goat<br>5. Sheep
205.	Does this household have any of the following?		
	1. Electricity	2. Solar Power	
206.	**TRANSPORTATION:** Does this household own any of the following? (**CIRCLE ALL THAT APPLIES)**		
	1. Motor vehicle<br>2. Motorcycle	3. Bicycle<br>4. Canoe/ boat	5. Donkey
207.	**COMMUNICATION:** Does this household own any of the following? (**CIRCLE ALL THAT APPLIES)**		
	1. Radio<br>2. Television	3. Mobile phone<br>4. Fixed phone	5. Postal address<br>6. Email address
208.	**INFORMATION:** What is the household's main source of information? (**CIRCLE ALL THAT APPLIES)**		
	1. Radio<br>2. Television<br>3. Print Media	4. Post Mail<br>5. Hand Mail	6. Word of Mouth<br>96. Other (specify)
209.	Does this household own any other land?		
	1. Yes	0. No	90. Don't Know
210.	Does this household own the land on which the house sits?		
	1. Yes	0. No, why?<br>1. No, not paying rent (squatting)<br>2. No, not paying rent at the consent of the owner<br>3. No, pays rent<br>4. Other (specify)	
211.	How many acres/ hectares of land does this household own in total? ____		

212.	What is the main source of the household's livelihood?			
	1. Subsistence farming<br>2. Employment income	3. Business enterprise<br>4. Cottage industry	5. Property income<br>6. Family support	7. World Food Program support<br>96. Other (Specify) ____________

(DIRECTIONS FOR INTERVIEWER): THE SUBSEQUENT QUESTIONS ARE DIRECTED TO THE CAREGIVER

213.	Sex of caregiver		
	1. Male	2. Female	
214.	What is your age (years)? ________________________		
215.	What religion are you affiliated with?		
	1. Catholic<br>2. Protestant	3. Muslim<br>4. Seventh Day Adventist	96. Other (specify): ______________________

216.	What tribe do you belong to?					
	1. Banyole<br>2. Bagwere	3. Bagisu<br>4. Japaghola	5. Basoga<br>6. Itseso	7. Baganda<br>8. Basamia	9. Banyankole<br>10. Acholi	96. Other (specify): ______________________

217.	Have you ever attended school?
	1. Yes<br>**IF YES, ASK:** What is the highest level of education you have achieved? / 0. No
	1. No education<br>2. Primary incomplete<br>3. Primary complete<br>4. Secondary incomplete<br>5. Secondary complete<br>6. Post Secondary (technical)<br>7. Post Secondary University<br>90. Don't know
218.	Have you done any work in the last 12 months for a wage?
	1. Yes / 0. No
219.	Apart from your <u>own</u> housework, are you currently working for a wage?
	1. Yes / 8. No
220.	What is your occupation? That is, what kind of work do you mainly do?
	1. Housewife<br>2. House keeper<br>3. Peasant farmer<br>4. Petty trader<br>5. Shop keeper<br>6. Unskilled labourer<br>7. Professional<br>8. Unemployed<br>96. Other (specify) ____________________

INTERVIEWER'S OBSERVATIONS: TO BE FILLED IN AFTER COMPLETING INTERVIEW

RECORD FINISHING TIME ____

COMMENTS ABOUT RESPONDENT:

____

____

____

COMMENTS ON SPECIFIC QUESTIONS:

____

____

____

____

SUPERVISOR'S OBSERVATIONS:

____

____

____

____

NAME OF THE SUPERVISOR: ____ DATE: ______________________________
