# Supplementary material for: Caregivers’ treatment-seeking behaviors and predictors of whether a child received an appropriate antimalarial treatment: a household survey in rural Uganda
Source: BMC Infect Dis. 2016 Sep 6;16(1):478. doi: 10.1186/s12879-016-1815-5 (PMC5012054; doi:10.1186/s12879-016-1815-5)
Supplement: Supplementary file 3 [file 12879_2016_1815_MOESM3_ESM.docx]

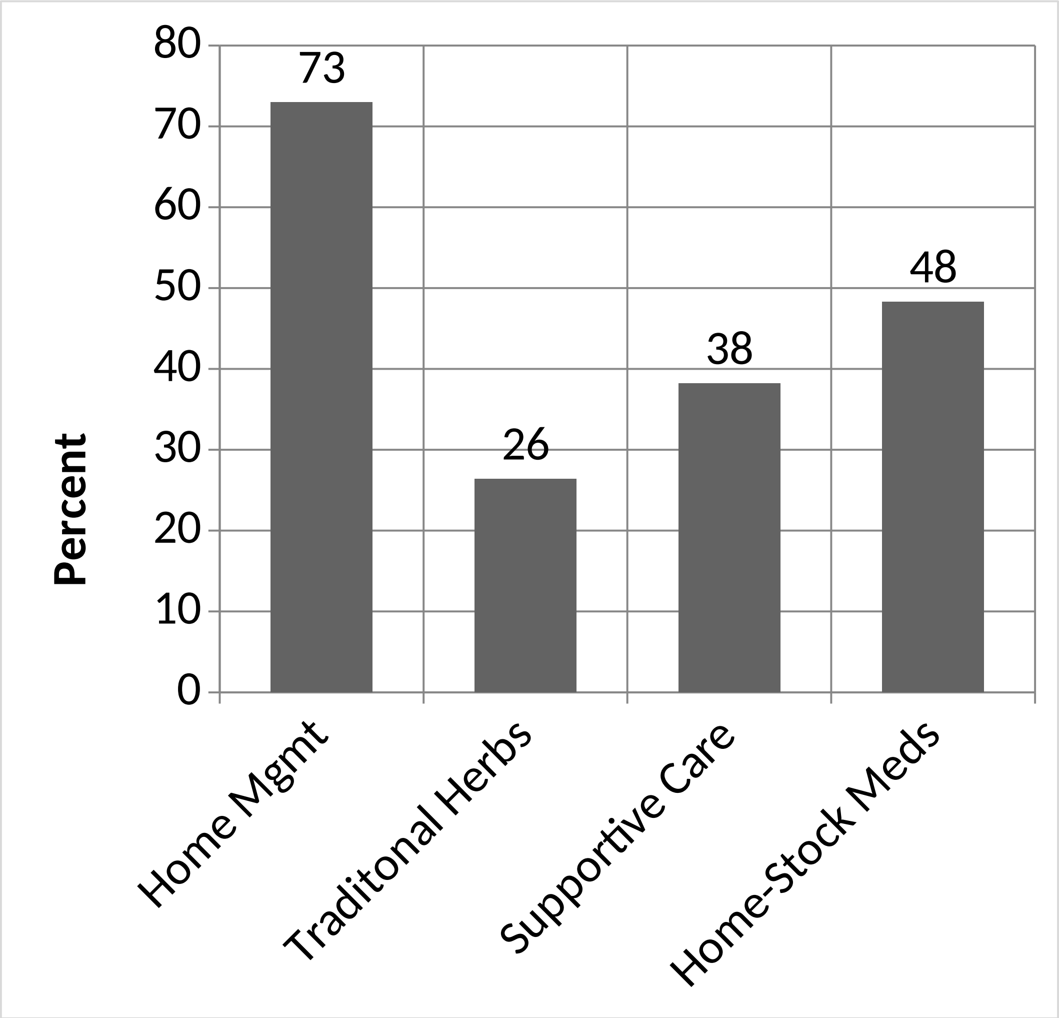

Percent
80
70
60
50
40
30
20
10
0
73
26
38
48
Home Mgmt
Traditonal Herbs
Supportive Care
Home-Stock Meds
