# Supplementary material for: Caregivers’ treatment-seeking behaviors and predictors of whether a child received an appropriate antimalarial treatment: a household survey in rural Uganda
Source: BMC Infect Dis. 2016 Sep 6;16(1):478. doi: 10.1186/s12879-016-1815-5 (PMC5012054; doi:10.1186/s12879-016-1815-5)
Supplement: Supplementary file 4 [file 12879_2016_1815_MOESM6_ESM.docx]

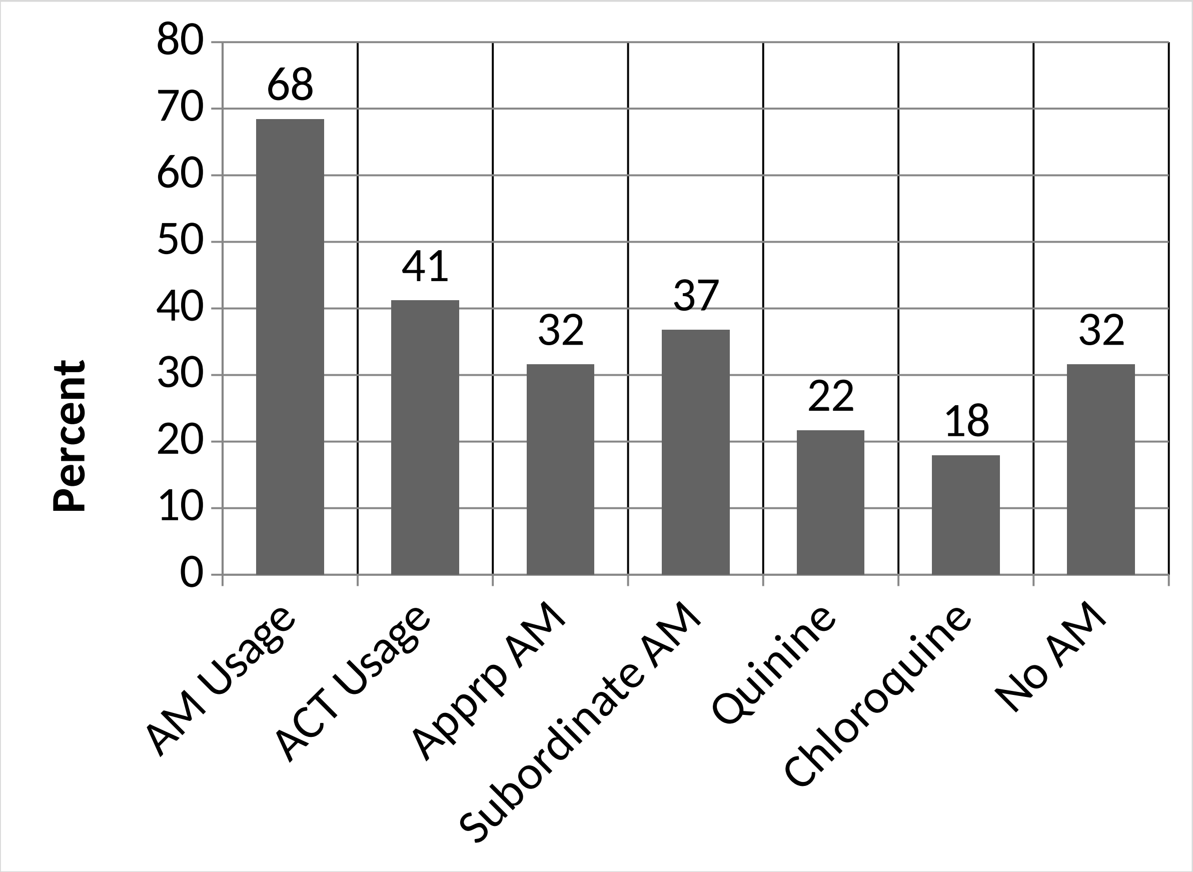
80
70
60
50
40
30
20
10
0
Percent
68
41
32
37
22
18
32
AM Usage
ACT Usage
Apprp AM
Subordinate AM
Quinine
Chloroquine
No AM
